# Gut Microbiomes of Indian Children of Varying Nutritional Status

**DOI:** 10.1371/journal.pone.0095547

**Published:** 2014-04-24

**Authors:** Tarini Shankar Ghosh, Sourav Sen Gupta, Tanudeep Bhattacharya, Deepak Yadav, Anamitra Barik, Abhijit Chowdhury, Bhabatosh Das, Sharmila S. Mande, G. Balakrish Nair

**Affiliations:** 1 Bio Sciences R&D, TCS Innovation Labs, Pune, India; 2 Center for Human Microbial Ecology, Translational Health Science and Technology Institute, Gurgaon, India; 3 Society for Health and Demographic Surveillance, West Bengal, India; 4 Department of Hepatology, School of Digestive and Liver Diseases, Institute of Post Graduate Medical Education and Research, Kolkata, India; Emory University School Of Medicine, United States of America

## Abstract

**Background:**

Malnutrition is a global health problem affecting more than 300 million pre-school children worldwide. It is one of the major health concerns in India since around 50% of children below the age of two suffer from various forms of malnutrition. The gut microbiome plays an important role in nutrient pre-processing, assimilation and energy harvest from food. Consequently, dysbiosis of the gut microbiota has been implicated in malnutrition.

**Methodology/Principal Findings:**

Metagenomics approach was adopted to investigate the gut microbiome sampled from 20 rural Indian children with varying nutritional status. The changes in the abundances of various taxonomic and functional groups were investigated across these gut microbiomes. A core set of 23 genera were observed across samples, with some showing differential abundances with varying nutritional status. One of the findings of the current study is the positive/negative associations of specific taxonomic and functional groups with the nutritional status of the children. Notable alterations in the architecture of the inter-microbial co-occurrence networks were also observed with changes in nutritional status. A key example is the clustering of potentially pathogenic groups into a distinct hub in severely malnourished gut. Our data does not demonstrate causality with the microbiome patterns that we observed, rather a description of some interesting patterns, whose underlying mechanism remains to be uncovered.

**Conclusions:**

The present study envisioned interrelationships between the pattern of gut microbiome and the nutritional status of children. The cause of this pattern needs to be explored. However, insights obtained from the present study form the basis for further metagenomic investigations on larger population of children. Results of such studies will be useful in identifying the key microbial groups that can be utilized for targeted therapeutic interventions for managing severe acute malnutrition.

## Introduction

In its journey down the human alimentary canal, food is intercepted by trillions of microbes (10^13^–10^14^) residing in the human gut. The human gut microbiome, collective genomes of all the microbes residing in the gastrointestinal tracts, provides several metabolic functions that are not encoded in our own genome. Examples of these functions include fermentation of dietary polysaccharides, anaerobic metabolism of proteins and peptides, biosynthesis of vitamins, absorption of ions and regulation of a number of host metabolic pathways [1]. These functions facilitate the pre-processing of dietary nutrients and efficient harvest of dietary energy for the host. Aberrations in the composition of the gut microbiome are thus likely to affect the nutritional status of the hostand lead to over/under-nutrition.

Malnutrition is a major health problem in developing countries and is characterized by symptoms like delayed growth, deficiencies in vital nutritional components, namely proteins, vitamins, minerals, essential fatty acids and others. Malnutrition can be broadly attributed to lack of food, and inability to successfully digest and use food that is available. Around 314 and 258 million children under the age of five in low and middle income countriesare estimated to suffer from stunting and wasting respectively [2]. Poor nutrition accounts for more than one-third of child deaths worldwide [3] and associated with half of all child deaths in India [4]. An estimated 42% and 58% of children in India (under the age of two) suffer from stunting and wasting respectively [5]. Linear growth retardation and low weight (for height or age) in children in developing countries like India cannot be attributed to food insecurity alone. Sub-clinical conditions like tropical/environmental enteropathy, widely prevalent in developing countries,have been reported to be responsible for nutrient malabsorption [6], [7]. A few recent studies have also indicated the association of the gut microbial community with malnutrition [8]–[10]. Dysbiosis of the gut microbiomeis likely to decrease the efficacy of nutritional interventions aimed at improving growth, thereby rendering them ineffective.

The gut microbiomes are observed to be heterogeneous across populations [11], [12]. Although previous studies have focused on the characterization of gut microbiota sampled from individuals belonging to diverse geographies, similar investigations on the Indian population are currently limited. Given the environment and unique socio-economic-cultural set up within India, the gut microbiome of the Indian population is expected to be different from those reported for other geographies.

Three public health indicators are commonly used for quantifying the extent of malnourishment in children [13]–[15]. These are: (1) Stunting (short height for age); (2) Wasting (low weight for height) and; (3) Underweight (low weight for age). Although a few studies have indicated the association of gut microbiota with malnutrition, the question of how gut microbial community structure changes with nutritional status remains unanswered till date. In order to obtain insights into this aspect, we have analyzed the gut microbiome from twenty Indian children of varying nutritional status (ranging from healthy to severe acute malnutrition).

## Materials and Methods

The details of the 20 subjects considered for this study, along with the methodology for sample collection, DNA extraction, pyrosequencing and quality filtering of sequenced metagenomic reads are described in Supporting Information S1 in File S1. All data were submitted to the NCBI Sequence Read Archive (SRA, http://www.ncbi.nlm.nih.gov/Traces/sra) with the following accession numbers SRR1067674, SRR1087919, SRR1068216, SRR1067721, SRR1068217, SRR1068218, SRR1068219, SRR1068596, SRR1068597, SRR1067720, SRR1067719, SRR1087910, SRR1087911, SRR1067716, SRR1087913, SRR1087914, SRR1087915, SRR1087916, SRR1067717, SRR1087918. Data was anonymized immediately after collection of the samples. The study was approved by the institutional review board of the Society for Health and Demographic Surveillance. Written informed consent was obtained from parents of the children in presence of another third person. The process of obtaining consent was not documented. The institutional review board had approved the procedurefor obtaining consent.

The nutritional status of each of the 20 children considered in this study was quantified using the WHO recommended (2009) three nutritional Z-scores namely, height for age (referred to in this study as Z-score1); weight for age (referred as Z-score2) and weight for height (referred as Z-score3). The formulae used for the calculation of these Z-scores, as recommended by WHO [15], are provided in Table S1 in File S1. Further, in order to obtain an overall measure of the nutritional status of these children, a cumulative Z-score was computed as the sum total of the above mentioned three Z-scores and was referred to as ‘Cumulative Nutritional Index’.

Based on the cumulative nutritional index, the 20 gut metagenomes were divided into three groups. These groups were (a) apparently healthy (AH) (or mild malnourished) with cumulative nutritional indices greater than −6, (b) borderline malnourished (BL) (−9<cumulative nutritional indices <−6) and (c) severely malnourished (SM) (cumulative nutritional index <−9).

### Taxonomic classification of metagenomic sequences

A database containing taxonomic information on known bacterial groups was created from the available data from the fully sequenced genomes at the NCBI database as well as from the bacterial/archaeal genomes sequenced as part of the Meta-HIT and the Human Microbiome Project consortiums. The database contained 2352 fully/partially sequenced bacterial genomes. Reads in each metagenome were subjected to BLASTN search [16] against this database (with thresholds of identity greater than 65%, query coverage >70% and minimum alignment length >75 base pairs). Subsequently, the reads were taxonomically classified using DiScRIBinATE [17]. DiScRIBinATE is a similarity-based taxonomic classification method that uses a two-step approach. Based on the alignment parameters (between a hit and its corresponding BLAST hit), the method first identifies an appropriate taxonomic level the read has to be assigned. In this study, the thresholds of alignment parameters used by Arumugam*et al.* [18] were utilized (by DiScRIBinATE) for identifying the appropriate taxonomic level. In the second step, DiScRIBinATE uses a bit-score/distance based approach to finally assign the read to a taxonomic group.

### Profiling the abundance of different taxonomic groups in the gut microbiomes

The abundances of different taxonomic groups (at the level of genus, family, order, class and phylum) in each gut microbiome were obtained by normalizing the number of reads assigned to the group in the corresponding gut metagenome by the number of sequenced reads in the entire metagenome [18]. The normalized abundance values of the taxonomic groups obtained were subsequently ranked across the 20 samples.

In order to identify taxonomic groups having positive or negative influence on nutritional status, Spearman rank correlations were computed between the ranked abundances of different groups in each metagenome and the cumulative nutritional index of the corresponding child. Taxonomic groups showing statistically significant correlations with at least one Z-score measure were suggested to influence the nutritional status of the children. The statistical significances of these associations were judged using the ReBoot Method [19]. The power associated with the correlation values was computed using the arctanh transformation method [20]. Additionally the multivariate analysis of taxonomic profiles of the 20 gut microbiomes were also performed using Principal Component Analysis (PCA) and Principal Least Square Discriminant Analysis (PLS-DA).

In order to identify taxonomic groups that were specifically over-abundant in one of the three groups of microbiomes (AH, BL or SM), the abundances of the microbiota across the three groups were compared, using both parametric (ANOVA) and non-parametric (Kruskal-Wallis-H) tests. Furthermore, to reduce the chances of Type I error, multiple test corrections were performed using the Benjamini-Hochberg FDR method. Given the lower sample size in the current study, this method was chosen over the Bonferroni method since the power of Bonferroni corrections (for datasets with limited sample size) is usually low and is likely to miss out on bona fide relationships. Further, Tukey-Kramer's test was used for post-hoc analysis of difference of means (for the relationships with significant p-values) in ANOVA. This method considers all possible pairs of means while controlling the family-wise error rate (i.e., accounting for multiple comparisons). The Effect size (measured by η^2^), which can be defined as the proportion of variance associated with or accounted for each of the main effects, interactions, and error in an ANOVA study, was also measured.

### Profiling the abundances of different functional categories in the gut microbiomes

Sequences in each metagenome were first subjected to a BLAST search (with thresholds of identity >65% and query coverage >90%) against the eggNOG database [21]. Based on the hits obtained, these sequences were assigned to different Clusters of Orthologous Groups (COGs) and subsequently to higher level functional categories.

The relative abundances of the different COG groups in each gut microbiome were obtained by dividingthe number of reads assigned to the group in the corresponding gut metagenome by the metagenome size. These abundance values were then rank normalized across the 20 samples.

In order to identify functional groups showing positive or negative associations with nutritional status, Spearman Rank correlations were calculated between the abundances of functional groups in each metagenome and the nutritional indices of the corresponding child. The COG groups showing significant positive or negative correlations (with P<0.05) were further analyzed.

The abundances of the various carbohydrate active enzyme families [22] as well as virulent factors [23] were then profiled across the 20 gut microbiomes. The details of the methodology adopted are provided in Supporting Information S2 in File S1.

### Identification of networks of co-occurring genera

For the metagenomes under study, pairwise correlations betweenthe rank normalized abundances of different genera were first obtained. Subsequently, pairs of genera having a correlation coefficients of P-value less than 0.05 (obtained using the ReBoot method) were linked by edges. Using this strategy, networks of co-occurring genera were generated and visualized using Cytoscape [24]. Finally various graph properties of the generated networks were computed using the Network-Analyzer plugin of Cytoscape. These properties included average number of neighbors, average path length, clustering co-efficient, network centralization, network density, network heterogeneity, number of nodes and shortest path. The networks of co-occurring genera (with P<0.01) were obtained separately for each of the three groups of children (AH, BL and SM). In order to study the transitions of the network architecture of co-occurring genera across the nutritional indices, the above mentioned graph properties were also calculated on a continuously evolving landscape, across varying nutritional indices, rather than as three discrete snapshots (corresponding to AH, BL and SM). For this purpose, a ‘sliding window’ approach was used. The 20 gut microbiomeswere first arranged in increasing order of their cumulative nutritional indices. Subsequently, 14 overlapping windows, each containing seven microbiomes,were considered. Pairwise correlation coefficients between the abundances of various genera were computed for each window. For each of the 14 windows, networks of co-occurring genera were then built following the above described procedure. The variations in the various graph properties werethen correlated with the average nutritional indices of the microbiomesin each of these windows.

## Results

### Anthropometric information and metadata of the subjects

The anthropometric data along with the sample metadata corresponding to the 20 children are provided in Table S2 in File S1. The overall variations in the three Z-scores (Z-score1, Z-score2 and Z-score3) corresponding to the nutritional status of the children constituting the study population were observed to range from −0.63 to −4.16, −1.07 to 4.32 and, 1.6 to −3.10, respectively. The above distribution indicates that the nutritional status of the children ranged from mild (i.e. apparently healthy) to severe malnourishment [14]. Cumulative Z-score ranged from −11.58 to −2.18. Overall the sizes of the gut metagenomes (in terms of the amount of nucleotide base pair data per sample) varied from 391 to 791 Mbps.

### Taxonomic composition inthe gut microbiomes

Across the 20 microbiomes, 72% of the sequences (ranging from 61.8% to 86.6%) could be assigned at the phylum level (Table S3 in File S1). Approximately 36.2% of the sequences (ranging from 21.8% to 50.7%) could be assigned to taxa at the genus level (Table S3 in File S1), while only around 4.2% of the sequences (ranging from 1.4–12.4%) could be assigned at the species level.

**Figure 1:**
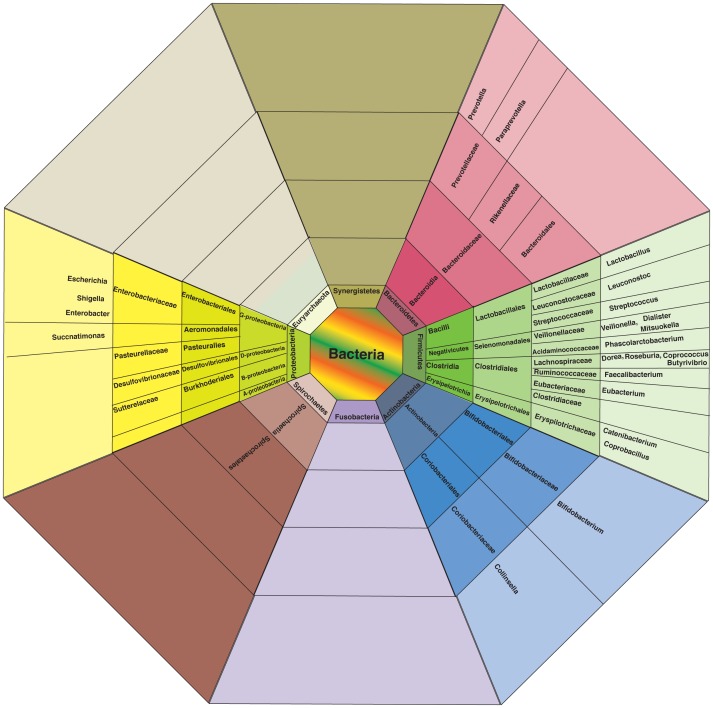
Distribution of taxonomic groups at various levels (including the core set of 23 genera) commonly present across at least 50% metagenomes with normalized abundance ratios of more than 0.01.

**Figure 2:**
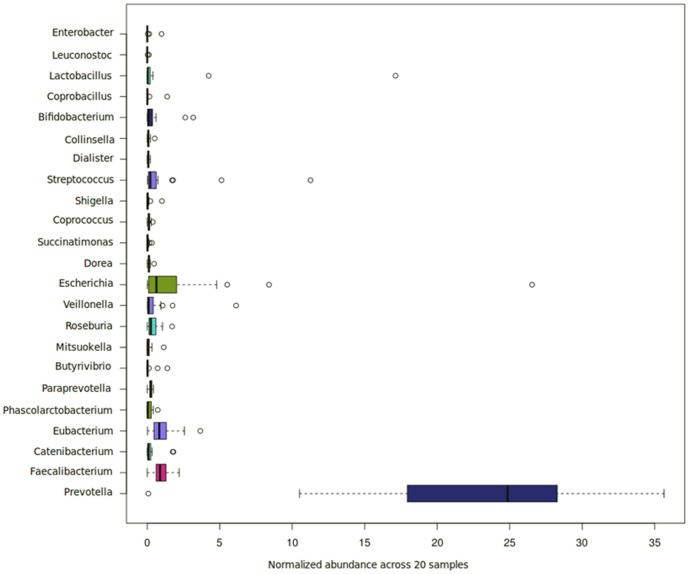
Variation of the normalized abundances (expressed as percentages) of the 23 core genera across the 20 gut metagenomes.

Eight phyla (Bacteroidetes, Firmicutes, Proteobacteria, Actinobacteria, Spirochaetes, Fusobacteria, Synergistetes and Euryarchaeota) were observed to be present across all 20 gut microbiomes (Table S4, S5, in File S1; Figure 1). Out of these, a core set of 23 genera belongingto four phyla (Bacteroidetes, Firmicutes, Proteobacteria and Actinobacteria) were observed to be present (with normalized abundance ratio greater than 0.01) in at least 50% (i.e. 10 out of 20) of the gut microbiomes (Figure 1). One of the key observations was the overall dominance of Prevotellaacross the metagenomes (Table S4, S5, in File S1; Figure 2), indicating that the majority of the gut microbiomes in the children under study were similar to the *Prevotella* enterotype gut microbial community previously studied by Arumugam*et al.* [18].

### Variation of microbial groups with nutritional status

**Figure 3:**
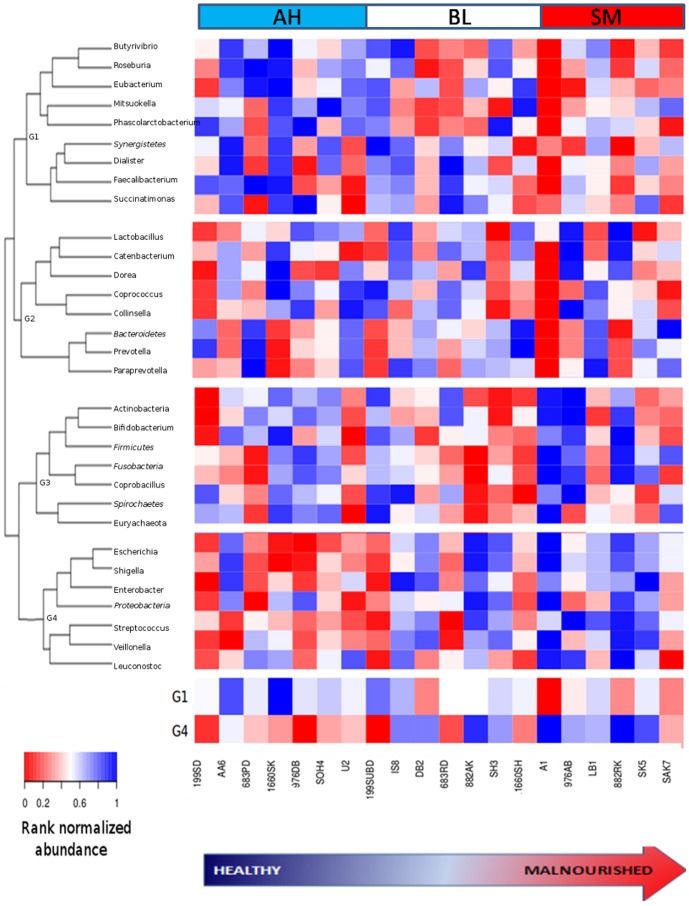
Variation of the rank normalized abundances of the 23 genera and 8 phyla (in italics) across the 20 gut metagenomes. The metagenomes are arranged in the bottom panel (from left to right) in decreasing order of their cumulative nutritional scores. The groups of microbiomes belonging to Apparently Healthy (AH), Borderline (BL) and Severely Malnourished (SM) children are also indicated. The taxa are arranged vertically on the left panel as a hierarchical tree based on the similarities in their abundance patterns. The four groups of taxa (G1–G4) with similar abundance patterns across the metagenomes are also demarcated. The cumulative rank normalized abundances of groups G1 (showing progressive increase with nutritional scores) and G4 (showing a progressive decrease with nutritional scores) across the 20 samples are provided in the bottom most panel.

Figure 3 shows the variations of the rank normalized abundances of the 23 core genera and the 8 phyla across the 20 gutmicrobiomes (arranged in order of their cumulative nutritional indices). A closer inspection reveals that the taxa could be divided into four groups (G1–G4) based on similarities in their abundance patterns. Taxa belonging to G4 were observed to have a progressive increase in their abundances with the decreasing nutritional index (Figure 3). The five genera belonging to this group namely, *Escherichia* (R = −0.59, P<0.032, Power at P-value<0.05: 0.85), *Streptococcus* (R = −0.70, P<0.019, Power at P-value<0.05: 0.97), *Shigella* (R = −0.62, P<0.04), *Enterobacter* (R = −0.75, P<0.032, Power at P-value<0.05: 0.97) and *Veillonella* (R = −0.80, P<0.005, Power at P-value<0.05: 0.99), wereobserved to have significant negative correlation with the nutritional index of the children (Table S6 in File S1). Taxa belonging to G2 and G3 do not show any significant correlation in their abundance patterns with the nutritional index of the study subjects.

The taxa belonging to the group G1 showed a progressive decrease in their rank normalized abundances with decreasing nutritional status (Figure 3). It was alsoobserved that several genera belonging to the group G1, namely *Roseburia* (R = 0.51, P<0.048, Power at P-value<0.05: 0.63), *Faecalibacterium* (R = 0.49, P<0.003, Power at P-value<0.05: 0.62), *Butyrivibrio* (R = 0.52, P<0.027, Power at P-value<0.05: 0.63) had significant positive correlations with nutritional index (Table S6 in File S1). Among the phyla, while Proteobacteria (belonging to G4) wasobserved to show a decrease with increasing nutritional status, Synergistetes (belonging to G1) was observed to havea positive correlation with nutritional status. Interestingly, the dominant genus in the analyzed gut microbiomes, namely *Prevotella*, was not observed to have significant correlations with nutritional indices. Unexpectedly, the probiotic genera like *Lactobacillus* and *Bifidobacterium* were also not observed to have any significant correlation with nutritional indices.

### Comparison of microbial groups across the three categories (AH, BL and SM)

**Figure 4:**
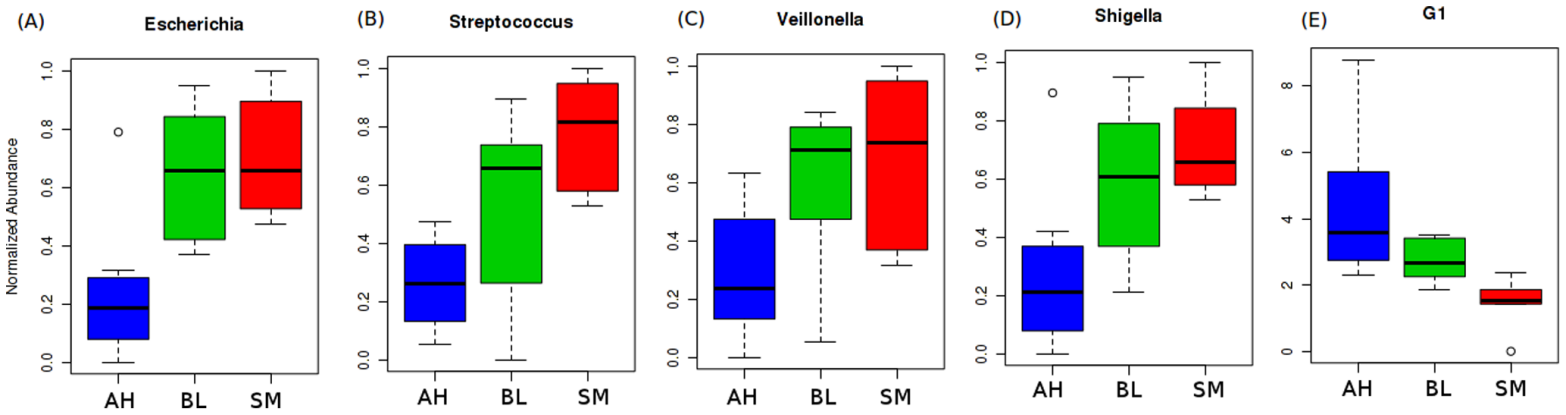
Box-plots showing the abundances of the taxonomic groups significantly differing across the three groups (AH, BL and SM) of gut microbiomes.

The results of the comparative analysis, using ANOVA, Kruskal-Wallis-H test (along with multi-test corrections), indicated five taxonomic groups that were significantly different across the three categories (AH, BL and SM) of microbiome (Figure 4, Table S7 in File S1). Several genera belonging to the group G4, namely *Escherichia*, *Streptococcus* and *Shigella*, were observed to be significantly over-abundant in the SM categories as compared to AH. These results are in line with those obtained using the correlation-based analysis (Table S6 in File S1, Figure 3). Further, although individual genera belonging to the group G1 did not show any significant differences across the three groups, the combined abundances of all the genera belonging to this group was observed to be significantly higher in the AH category (Figure 4, Table S7 in File S1). This result suggests that, not all members of a particular group of genera (having similar characteristics) may be required for conferring a specific phenotype. Multivariate analysis using PCA as well as PLS-DA also indicated a distinct separation between the AH and SM categories of microbiomes, with the BL categories of microbiomes interspersed in between (Figure S1 in File S1).

### Functional profiles ofthe gut microbiomes

**Figure 5:**
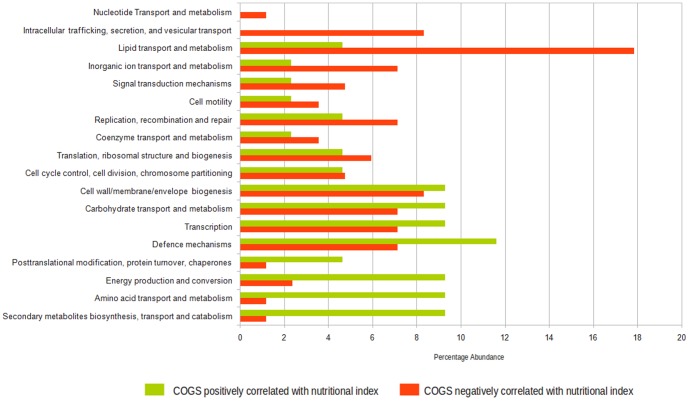
Percentage abundances of the different functional categories corresponding to the COGs identified either as positively correlated (shown in green) or as negatively correlated (shown in red) with nutritional indices.

64 and 112 COG groups were observed to have significant positive (ReBoot Z-value>1.97; P<0.05) and negative correlation (ReBoot Z-value<−1.97; P<0.05) with the cumulative nutritional indices, respectively (Tables S8 and S9 in File S1). These two COG groups were referred to as Positively Correlated (PC) and Negatively Correlated (NC) COG groups. The COGs belonging to the PC and NC groups were then mapped to their respective higher level functional categories. The relative abundances of the different functional categories in the two groups were then compared (Figure 5).

Several functional categories associated with nutrient uptake and metabolism was observed to be either over-represented or present only in the PC COG groups (Figure 5). These functional categories included secondary metabolites biosynthesis, transport and catabolism; energy production and conversion; amino acid transport and metabolism and carbohydrate transport and metabolism. The only exception to the above trend was lipid transport and metabolism which was observed to be over-represented in the NC COG group.

In contrast to the PC COG groups, the NC COG group was observed to have an over-representation of functional categories that have been associated with virulence and bacterial pathogenesis [8]. These included intracellular trafficking; secretion and vesicular transport; cell motility and inorganic ion transport and metabolism (Figure 5).

### Profiles of carbohydrate active enzyme families in the gut microbiomes

**Figure 6:**
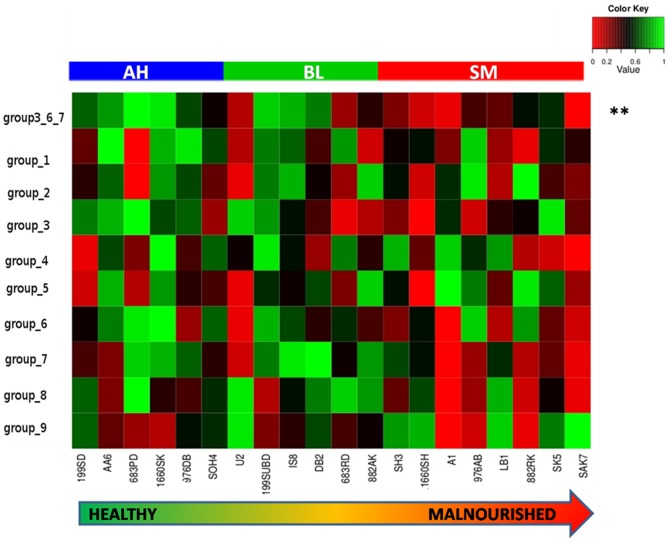
Abundance patterns of different groups of CAZyme families across gut metagenomes with varying nutritional status. The metagenomes are arranged in the bottom panelin decreasing order of their cumulative nutritional scores. The groups of microbiomes belonging to Apparently Healthy (AH), Borderline (BL) and Severely Malnourished (SM) children are also indicated. “**”indicates the variation of the cumulated abundances of members belonging to groups 3, 6 and 7.

The results of the analysis indicated that none of the CAZyme families were significantly correlated with the cumulative nutritional index (Figure S2 in File S1). In order to evaluate whether groups of CAZyme families worked in tandem and showed significant correlation with the nutritional index, CAZyme families having similar abundance patterns were grouped together and analysed (Figure 6, Supporting Information S2 in File S1). The constituent CAZyme families within each of these groups are provided in Table S10 in File S1.

It was observed that CAZyme families belonging to groups 3 and 7 had significant (P<0.05) positive correlations with at least one of the nutritional indices (Figure 6, Table S11 in File S1). While most of the CAZyme families belonging to group 3 are known for degrading complex plant carbohydrates, those belonging to group 7 are mostly peptidoglycan degraders. In addition, group 6 was observed to have marginal positive correlations (P<0.10) with Z-score2 as well as with the overall nutritional index. CAZyme families belonging to group 6 are chiefly responsible for degradation of complex plant carbohydrates. When the CAZyme families belonging to groups 3, 6 and 7 were combined together, their cumulated abundances were found to have an even stronger correlation with the overall nutritional index (R = 0.583, P<0.007). Interestingly, none of the groups were observed to have significant negative correlation with nutritional indices.

### Profiles of virulence factors in the gut microbiomes

The number of virulence factor homologs detected in the microbiomes was observed to show a progressive increase with decreasing cumulative nutritional index (R = −0.498, P<0.02) (Figure S3 in File S1). Thus, the presence of higher number of virulence genes in children with lower nutritional index indicates the presence of more pathogenic species.

### Networks of co-occurringgenera and nutritional indices

The symptomatic characteristics of the gut environment depend not only on the presence of specific microbial groups but also on the inherent inter-microbial co-occurrence networks present therein. The network of co-occurring genera, obtained for the three groups of gut microbiomes (AH, BL and SM), were observed to be characterized by distinct network architecture.

**Figure 7:**
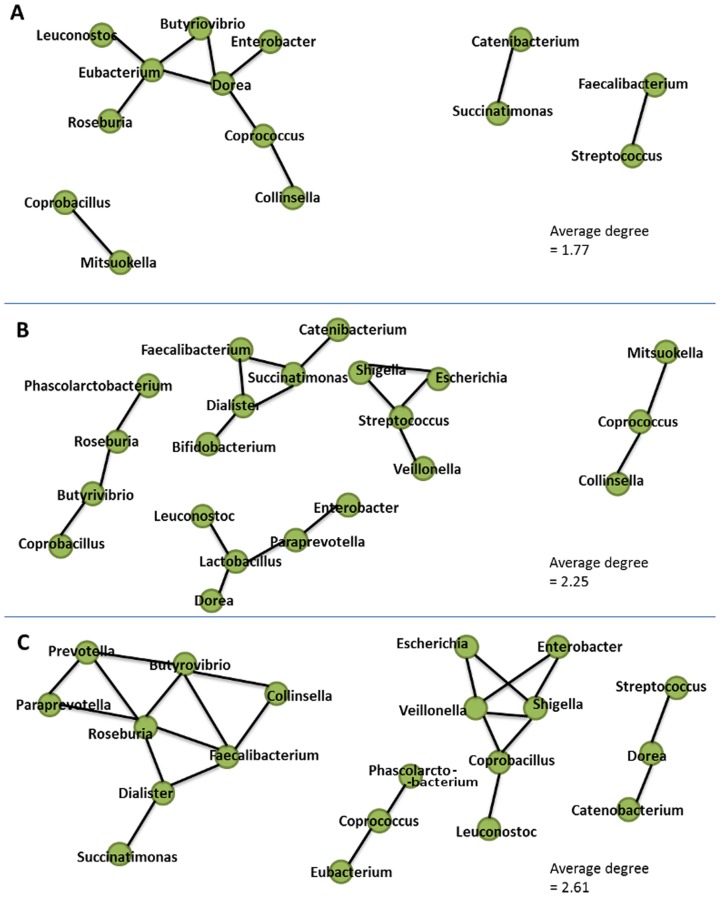
Genera co-occurrence networks obtained for gut microbiomes from (A) Apparently Healthy, AH, (B) Borderline, BL and, (C) Severely Malnourished, SM children.

The co-occurrence network of the AH group of metagenomes were characterized by four distinct connected groups of genera. Interestingly, although genera belonging to the groups G1 and G4 were observed to have contrasting trends in their abundance patterns, some of themshowed strong positive associations amongst each other (Figure 7). For example, the genus *Streptococcus* (belonging to the group G4) was observed to be associated with beneficial genera like *Faecalibacterium* (belonging to group G1). Similarly the genera *Enterobacter* (belonging to G4) was observed to be associated with genera *Eubacterium*, *Roseburia* and *Dorea* (from group G1).

Distinct changes were observed in the genera co-occurrence network with a progressive decrease in the nutritional status. For example, in the borderline (BL) group of metagenomes, genera belonging to the group G4 (namely, *Esherichia*, *Shigella*, *Streptococcus*, *Veillonella*) were observed to be placed together in a single connected hub. Furthermore, the average node density of the co-occurrence networks obtained for the borderline metagenomes was also observed to be higher as compared to that obtained for the apparently healthy metagenomes. This probably indicates that with a progressive decrease in nutritional status, there may be an increasing degree of functional interdependence among genera (especially those belonging to the group G4).

The increasing interdependence among the genera is observed to be even more pronounced in the co-occurrence networks obtained for the severely malnourished group of metagenomes. The networks of co-occurring generain the severely malnourished (SM) microbiomes were observed to contain two dominant hubs of genera. While one hub was primarily constituted by the likely pathogenic genera (e.g. *Shigella*, *Escherichia* and *Enterobacter*) belonging to the group G4, the second hub was constituted by the commensal and the beneficial genera (e.g. *Roseburia*, *Butyrivibrio* and *Faecalibacterium*), belonging to G1, in addition to *Prevotella* and *Paraprevotella*. These results suggest that the gut microbial community in SM group is likely to be characterized by the presence of two (functionally opposing) groups of genera. The average node degree within these two hubs were also observed to be high (28 nodes for 20 genera; average node degree: 2.61), indicating a strong inter-dependency within the members in each group.

**Figure 8:**
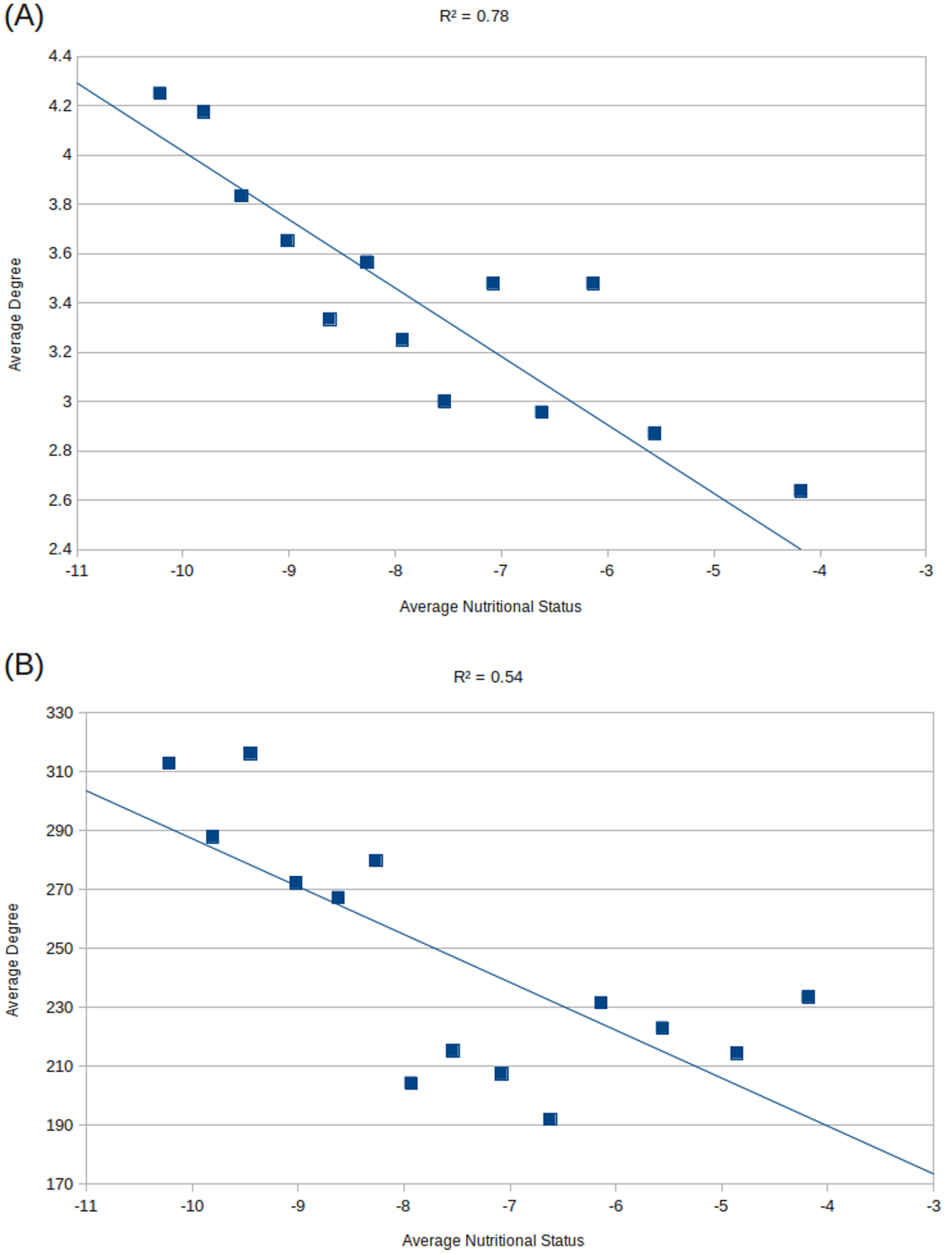
Variations of graph properties (of the genera co-occurrence networks obtained for the overlapping groups of metagenomes) namely, (A) Average number of neighbors and, (B) Shortest path with the average nutritional index.

Analysis of graph properties of co-occurrence networks obtained for the 14 overlapping groups of metagenomes (described in the Methods section) identified the average number of neighbors to have significant negative correlation with the nutritional status (Figure 8a, Figure S4 in File S1) indicating that with an increase in the nutritional status, the inter-dependence amongst microbial groups shows a progressive decrease. This result is in line with that obtained for the three discrete groups of metagenomes (Figure 7). Similarly, Figure 8b shows the variation of the average number of neighbors across COG co-occurrence networks obtained for the groups of gut microbiomes with progressively increasing status. The average number of neighbours obtained in each of the COG co-occurrence networks also showed a similar pattern to that obtained using the microbial genera. This further indicates a likely decrease in functional inter-dependence with an increase in nutritional status.

## Discussion

The onset and progression of malnutrition has been attributed to a variety of causal factors. These include inadequate nutrient intake, prolonged enteric infections due to sub-standard hygiene, metabolic disorders and the variations in the taxonomic and functional composition of the gut microbiota. Previous studies have provided the early snapshots of the taxonomic and functional dysbiosis in the gut microbiome of malnourished children [8], [9]. A separate study by Smith *et al.* [10] implicated the gut microbiome as a causal factor in kwashiorkor, an enigmatic form of severe acute malnutrition. These studies have provided the first insights into the role of the gut microbiome in this global health problem. Several pathogenic bacteria, typically associated with enteric infections, were reported in the gut of a malnourished child [8] Such aberrations in gut microbiome are expected to lead to sub-clinical disorders like inflammation and nutrient malabsorption. Infection has been known to adversely affect nutritional status and also malnutrition can predispose children to infections [25]. The current study has attempted to deepen these insights further by performing a comprehensive investigation of gut microbiomes obtained from a larger population of children from rural India with varying nutritional status.

The present study indicates that the impaired nutritional status is not only due to the abundances of likely pathogenic microbial groups, but also a result of depletion of several commensal genera. These genera, namely, *Roseburia*, *Faecalibacterium*, *Butyrivibrio*, *Eubacterium* and *Phascolarctobacterium*, have been suggested to have positive influence on the nutritional status of the children. These genera have earlier been reported to be a part of the normal commensal microflora of the human gut [26]–[32]. Several studies have indicated the beneficial and probiotic properties of these genera in either nutrition harvest from the food or in maintaining the immune stability of the gut. For example, genera like *Roseburia* and *Butyrivibrio* are known producers of short chain fatty acids (SCFA) in the intestine [27], [28]. The production of SCFA from the dietary fibers has been reported to be crucial factor for improved harvest and intake of carbohydrates [29], [30]. Furthermore, genera like *Faecalibacterium*, *Roseburia* and *Eubacterium* have been shown to possess anti-inflammatory properties [27]–[32]. Studies have suggested that depletion of species belonging to these genera could be associated with low grade inflammation and onset of inflammatory bowel diseases [31], [32]. Inflammation has also been associated with malabsorption of nutrients and subsequent decline in the nutritional status [33].

The present study revealed that certain functional categories (COG groups) are positively/negatively correlated with nutritional status. The identified positively correlated functional groups are likely to aid in better utilization of the nutrients in the gut, thereby helping in maintenance of the overall well-being of the child. On the other hand, the negatively correlated functional groups may initiate the infection process which might indirectly lead to malabsorption of nutrients. The higher number of virulence genes identified in the children with lower nutritional index further corroborates this finding.

A key finding of the current study is the identification of three groups of carbohydrate active enzymes (called CAZymes) families (in the gut microbiomes) showing positive associations with the nutritional status of the children. These groups were observed to be dominated by CAZyme families that preferentially degrade peptidoglycans and complex plant carbohydrates. Dietary fibers, chiefly constitutedby complex carbohydrates, cannot be completely degraded by enzymes coded by the human genome. These complex carbohydrates can only be degraded by the enzymes encoded by the gut microbiota. The human host can harvest energy from such recalcitrant carbohydrates only after bacterial enzymes have pre-processed them. Such ‘energy harvest’ in the gut becomes particularly crucial for subjects with lower nutritional status.

Identification of distinct changes in genera co-occurrence networks with progressive decrease in the nutritional status of the children is a key finding in the current study. The results indicate that with decrease in the nutritional status, there is a likely increase in functional interdependence among the various microbes residing in the gut. It was also interesting to observe the associations of probable pathogenic groups with potentially beneficial ones in healthy gut microbiomes. Clustering of potentially pathogenic groups into a distinct hub in severely malnourished gut probably suggests how these groups may help each other in a cooperative manner to a create an imbalance in microbial populations. This in turn may lead to the creation of milieu that is progressively detrimental in maintaining the nutritional homeostasis of the gut. Functional associations within microbial groups may modulate specific functional behaviors of its constituent members in terms of their expressed genes and proteins. Thus, the observed changes in the architecture and composition of networks of co-occurring microbial groups under different nutritional settings add further insights into the gut microbial environments associated with the nutritional status. The functional interdependence of the network could be used in future for therapeutic interventions to address the challenge of clinical management of severe acute malnutrition. The network analysis suggests that one can modulate the gut microbiome by disrupting certain key players (genera) in order to achieve conditions that could result in the regression of the disease phenotype.

Considering the pilot nature of the study and the limited sample size, the results obtained in the study should be interpreted with due caution. However, the present findings open up an interesting premise which furthers our understanding of the role of gut microbiota in causation of malnutrition under nutrient limited conditions. Further, given that the geographical location, age and diet of most of the children (considered in the current study) were similar, it was assumed that the observed trends (of either increasing/decreasing functional interdependence) could provide a rough indication of the changes in the microbiome with varying nutritional status. However, in order to obtain a definitive picture of the underlying changes associated with either increase or decrease of nutritional status, a longitudinal study is absolutely necessary. We further acknowledge that ‘unacknowledged heterogeneity’ remains as there could be numerous genetic and environmental factors affecting the composition of the gut microbiome [12]. Additionally, other unmeasured epidemiological factors could be influencing our findings and this warrants replication in a larger population. It is virtually impossible to consider all the factors in a single analysis. However, we have tried to obtain samples from a similar age-geography-economy strata (all children from eastern part of India from a low economic strata which are supposed to be the key factors in influencing gut microbiota composition). Thus, we have tried to reduce the number of variables in our analysis as much as possible. We expect that, considering the pilot nature of the study, the results provide a rough picture of microbial community dynamics, at least at the basic level.

## Conclusion

The present study describes patterns wherein the gut microbiome varies in response to nutritional status. Although the biological bases of such patterns may be inferred to a certain extent, the universality and the biological mechanisms governing the occurrence of these patterns remains to be experimentally verified. Although limited by sample size and range of nutritional status of selected subjects, the present study opens up avenues for in-depth analyses of gut microbiomes on much larger populations belonging to diverse geographies as well as nutritional status. Such studies will help in better understanding the role of microbial groups in host nutrient assimilation and how they interact, which are evolving as important determinants of systemic conditions like malnutrition. Much comprehensive and well designed functional studiesare required for formulating a microbial basis of therapy for severe acute malnutrition.

## Supporting Information

File S1 **Combined supporting information.** Additional data including details of study population, sample collection, DNA extraction, pyrosequencing, quality filtering, abundances of carbohydrate active enzyme families and virulence factors in the gut microbiomes are provided in supporting information in File S1. Supplementary Figures and supplementary Tables are also available in File S1.
(PDF)
