# Supplementary material for: Gut Microbiomes of Indian Children of Varying Nutritional Status
Source: PLoS One. 2014 Apr 24;9(4):e95547. doi: 10.1371/journal.pone.0095547 (PMC3999041; doi:10.1371/journal.pone.0095547)
Supplement: File S1 — Combined supporting information. Additional data including details of study population, sample collection, DNA extraction, pyrosequencing, quality filtering, abundances of carbohydrate active enzyme families and virulence factors in the gut microbiomes are provided in supporting information in File S1. Supplementary Figures and supplementary Tables are also available in File S1. (PDF) [file pone.0095547.s001.pdf]

Supporting Information S1

Details of study population, sample collection, DNA extraction, pyrosequencing and quality filtering

Study population

In Birbhum district, West Bengal, India, the population demography and socio-economic characteristics, human development and health indices are typical of rural and agriculture dependent West Bengal. The Birbhum population project (BIRPOP), established in 2008, is a health and demographic surveillance system (HDSS; http://www.shds.in/about_us.html) that involves longitudinal observation and analysis in the context of a systematically organized population cohort of c.a. sixty thousand residents. To determine how malnutrition affects gut microbiome composition and function, 20 children of varying nutritional status (from apparently healthy to ones suffering from various anthropometric failure, for example, either wasted, stunted or underweight) were selected as study subjects from this population. All subjects were aged less than or equal to 60 months. They had no history of diarrhea or any acute respiratory infection or had consumed any antibiotics for a period of at least four weeks before fecal sample collection. Z-scores were calculated for each of these subjects before enrolling them in the study. Children with at least two of the three measured Z-scores lying above the level of median minus twice the standard deviation values were considered as healthy [1], and subjects having all three Z-score values below this were considered as malnourished. Subjects with at least two of the measured Z-score values falling below the level of median minus thrice the standard deviation and the third Z-score value below level of median minus twice the standard deviation were grouped as subjects with severe acute malnutrition.

Sample collection and DNA extraction

Faecal samples from 20 children under study were collected aseptically in sterile stool containers and transported to the laboratory in dry ice within 6-8 hours of collection. Total bacterial DNA was extracted from 200mg of each faecal sample using the QIAamp DNA Stool Mini Kit (Qiagen, USA) using the manufacturers protocol. To ensure maximum lysis of bacterial cells, samples were lysed by mechanical disruption using 0.1mm Zirconium beads and a mini bead beater (BioSpec Products, Inc., USA).

Quality assessment and quantitation of genomic DNA

The quality and DNA concentration of each of the samples were assessed using NanoDrop ND1000 spectrophotometer (Thermo Scientific, USA) and 0.8% agarose gel electrophoresis. Samples with OD_{260}/OD_{280} ratio $\geq$ 1.8, OD_{260}/OD_{230} ratio $\geq$ 1.9, DNA concentration between 250 to 500ng/μL and with no visible evidence of contaminating RNA or DNA degradation, were used for library preparation for whole genome sequencing.

Library preparation

DNA from each of the samples was subjected to Rapid Library preparation protocol for GS FLX sequencing by Titanium chemistry (Roche, USA). Briefly, about 1 μg of genomic DNA was nebulized by nitrogen gas at a pressure of 30 psi for 1 minute for obtaining DNA fragments of length 500-900 base pairs (bps). The nebulized DNA was purified by MinElute PCR Purification kit (Qiagen, USA), end polished with T4 DNA Polymerase, Polynucleotide Kinase and Taq DNA Polymerase (Roche, USA) by incubating at 25ºC for 20 minutes and 72ºC for 20 minutes. This was followed by ligation of library adapter oligonucleotides using DNA Ligase

(Roche, USA) and incubating at 25ºC for 10 minutes. The genomic DNA libraries thus obtained were then purified by Ampure XP (Beckman Coulter, USA). The DNA libraries were analyzed using DNA High Sensitivity Chip in 2100 Bioanalyzer (Agilent, USA) and quantified using Picogreen dye in QubitFluorometer(Invitrogen, USA) and NanoDrop.

Whole genome sequencing

About 140 x 10^7 DNA library molecules per sample were clonally amplified by emulsion PCR in ABI 9700 PCR systems (Invitrogen, USA), purified using REMe integration (Roche, USA) on Biomek 3000 (Beckman Coulter, USA) and pyrosequenced in pico-titre plates in GS-FLX Genome Sequencers (Roche, USA). For sequencing, one pico-titre plate was used per sample. Sequence reads in FASTQ format were obtained from SFF files usingsffinfo (GS-FLX) and a Linux script developed in-house. The sequence data generated per run were evaluated by FASTQC (http://www.bioinformatics.babraham.ac.uk/projects/fastqc/), using default parameters.

Quality filtering

In order to retain only the high quality reads in each metagenome, sequence reads having lengths less than 75 bps and at least one base with PHRAP quality scores less than 20, were removed from the metagenomic datasets.

Removal of host contamination

Sequences originating from host DNA were removed by first detecting probable host contaminating sequences using Eu-Detect [2] and then performing a BLASTN search (with

threshold identity >95% and query coverage >90%) of this subset against the Human Genome v19.

References

1. FAO (1992) World Food Supplies and Prevalence of Chronic Undernutrition in Developing Regions As Assessed in 1992. Rome: FAO, Rome.

2. Mohammed MH, Chadaram S, Komanduri D, Ghosh TS, Mande SS (2011) Eu-Detect: an algorithm for detecting eukaryotic sequences in metagenomic data sets. J Biosci 36: 709-717.

Supporting Information S2

Profiling the abundances of carbohydrate active enzyme families and virulence factors in the gut microbiomes

Putative homologs of carbohydrate active enzymes (CAZymes) present in the 20 gut microbiomes were identified by performing a BLASTN search of the metagenomic datasets against the CAZymes present in the CAZy database (http://www.cazy.org/.accessed on 2 August 2013) [1]. Sequences in each metagenome having the best BLAST hit (with e-value < 1e-06 and nucleotide identity ≥ 65%) were retained.

For each metagenome, the number of significant hits to each CAZyme family was first obtained. This number was subsequently divided by the number of reads in the metagenome and was represented in the form of an 'Abundance Matrix'. In order to remove the effect of sampling bias across the 20 gut metagenomes, the 'Relative Homolog Abundances' (RHA) of each CAZyme family was calculated as follows:

RHA= [(Number of CAZymes in a family in a given sample)/ (Total number of CAZymes in a given sample)] *100

For the purpose of comparison, the RHA of each CAZyme family was then cross-normalized across the 20 metagenomes and represented as Z-scores.A heat map representing the normalized RHA values across the different gut metagenomes was generated (using the heatmap2 module of R statistical package) in order to identify CAZyme families which were significantly correlated with the overall nutritional status. For generating the heat map, while the CAZyme families were clustered using Agglomerative Clustering Algorithm (Ward's Method) [2], the samples were arranged in the increasing order of overall nutritional index.

In order to obtain a better resolution for the heatmap, CAZyme families showing similar abundance profiles across the 20 microbiomes were clustered into groups. The RHA of each group was computed by cumulating the abundances of the CAZyme families within each group. For each group, the RHA values were subsequently rank normalized across the 20 microbiomes. These ranks were then range-scaled from 0 to 1. The range-scaled ranks were then plotted in the form of a heat map, with samples arranged in increasing order of the overall nutritional index and the groups of CAZyme families re-clustered using Ward's method [2].

In order to identify groups of CAZyme families that showed significant correlation (positive or negative) with nutritional indices, Pearson's Correlation coefficients were calculated between the range-scaled ranks and the nutritional indices of the children. Groups of CAZyme families with significant correlation coefficients (P-value < 0.05) were then identified.

Profiling the abundance of virulence genes in the gut microbiomes

Sequences corresponding to the homologs of virulence factors were downloaded from Virulence Factor (VF) database (http://www.mgc.ac.cn/VFs accessed on 2 August 2013) [3]. Similarity searches of sequences in each gut metagenome were performed using BLASTx against the database of VF homologs. In each metagenome, VFs having atleast 70% of their lengths covered in alignments (with identity >70% and query coverage >80%) were identified. The overall abundance of VFs in a given microbiome was then computed by normalizing the number of VFs detected in a given metagenome by the number of sequences in the corresponding metagenome.

References

1. Cantarel BL, Coutinho PM, Rancurel C, Bernard T, Lombard V, et al. (2009) The

Carbohydrate-Active EnZymes database (CAZy): an expert resource for Glycogenomics. Nucleic Acids Res 37: D233-238.

2. Joe H. Ward J (1963) Hierarchical Grouping to Optimize an Objective Function. Journal of the American Statistical Association 58: 236-244.

3. Chen L, Yang J, Yu J, Yao Z, Sun L, et al. (2005) VFDB: a reference database for bacterial virulence factors. Nucleic Acids Res 33: D325-328.

Supporting figure legends

Figure S1.

Multivariate analysis of the 20 gut microbiomes using (A) Principal Component Analysis (PCA) with both PC1 v/s PC2 as well as PC1 v/s PC3 and (B) Principal Least Square Discriminant Analysis (PLS-DA).

In (A), Apparently Healthy (AH), Borderline (BL) and Severely Malnourished (SM) gut microbiomes are denoted as Green triangles, Orange squares and Blue rounds, respectively.

In (B), Apparently Healthy (AH), Borderline (BL) and Severely Malnourished (SM) gut microbiomes are denoted as Red, Green and Blue dots, respectively.

Figure S2.

Abundance patterns of various CAZyme subfamilies across varying nutritional status. The parent node (under which all the child nodes are clustered) are marked with "*". The group numbers are demarcated in the right margin.

Figure S3.

Variation of abundance of virulence factors across groups of microbiomes with varying nutritional status. AH represents microbiomes from apparently healthy individuals, BL represents individuals with borderline malnourishment and SM represents severely malnourished individuals.

Figure S4.

Genera co-occurrence network for fourteen overlapping windows (1 to 14) of gut metagenomes with decreasing nutritional index.

Figure S1.

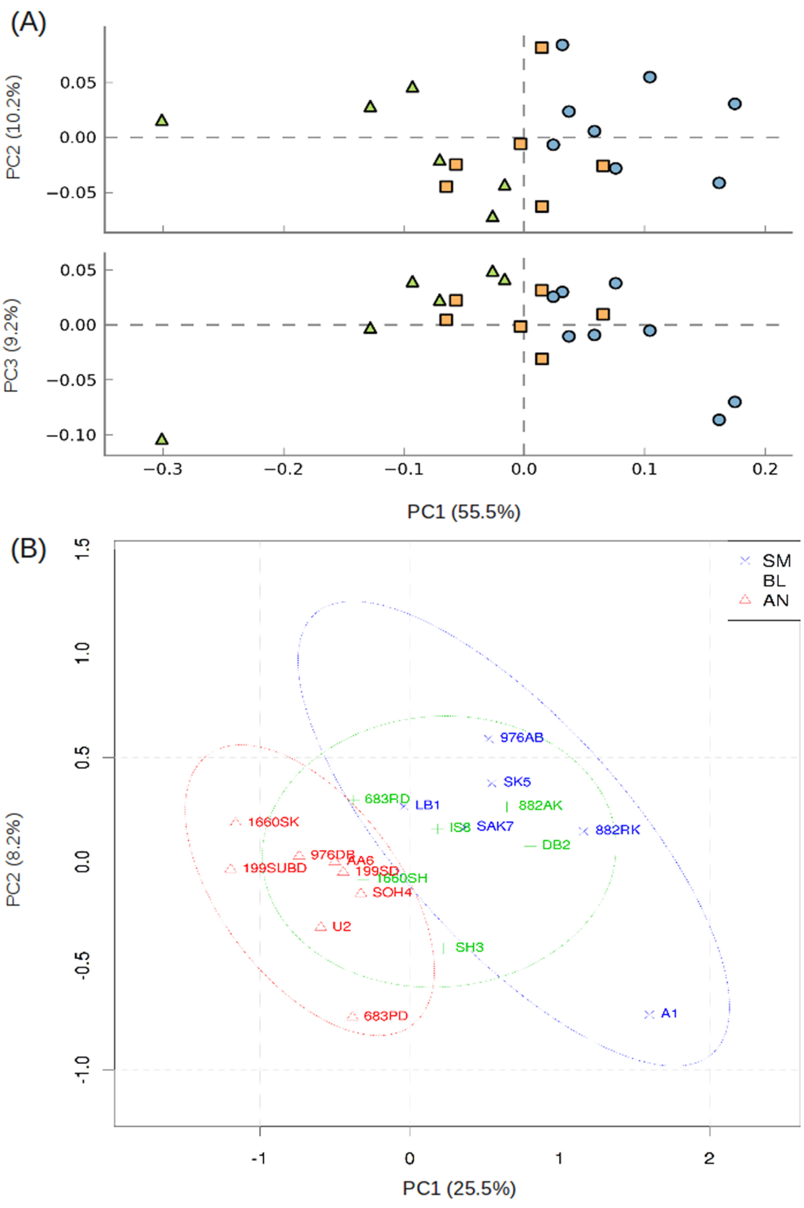
(A)
PC2 (10.2%)
PC3 (9.2%)
PC1 (55.5%)
(B)
SM
BL
AN
976AB
SK5
683RD
LB1
882AK
1660SK
IS8
SAK7
882RK
DB2
976DB
AA6
199SUBD
199SD
1660SH
SOH4
U2
SH3
683PD
A1
PC2 (8.2%)
PC1 (25.5%)

Figure S 2.

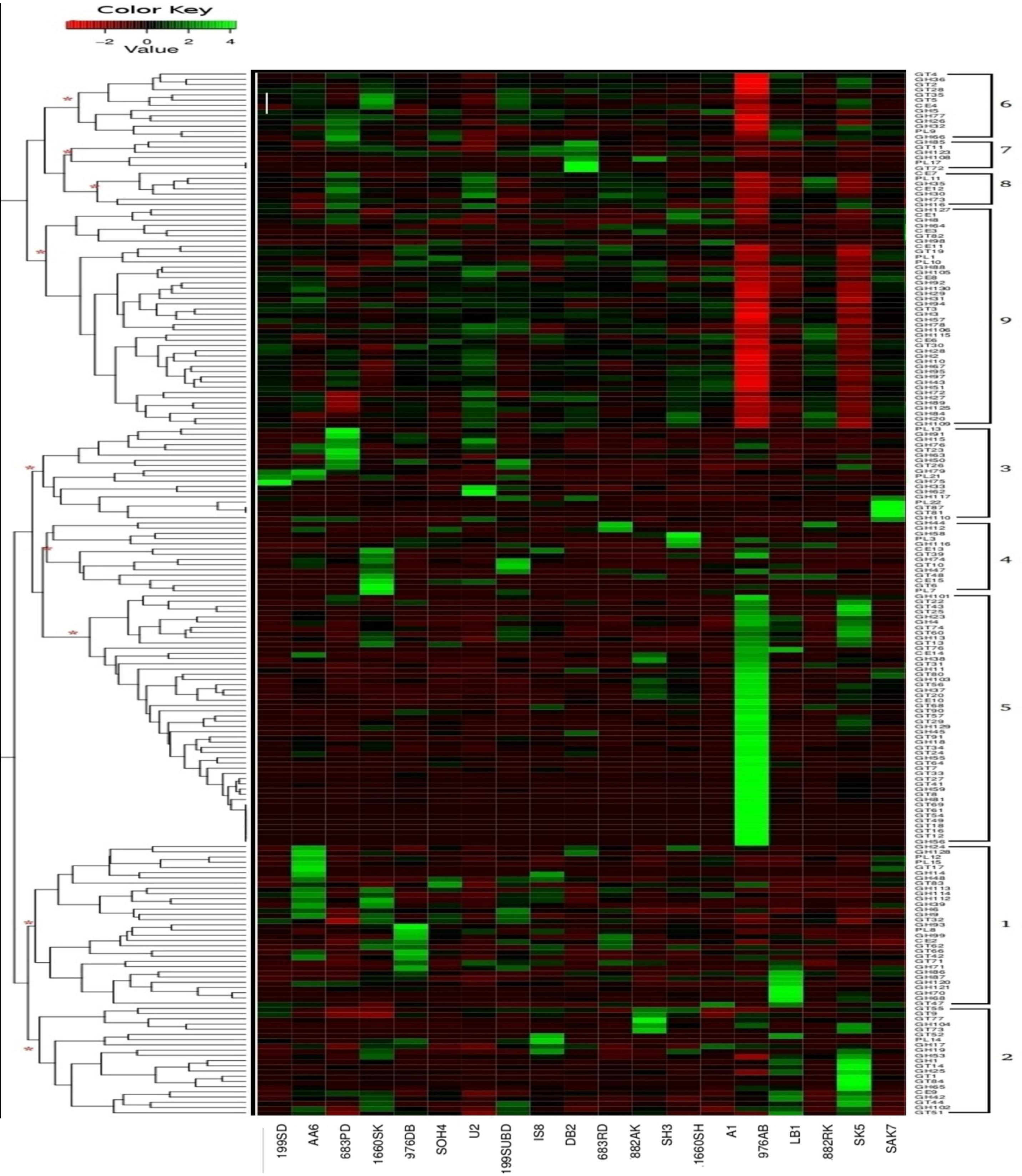

Color Key
-2 0 2 4
Value
6
7
8
9
3
4
5
1
2
199SD
AA6
683PD
1660SK
976DB
SOH4
U2
199SUBD
IS8
DB2
683RD
882AK
SH3
1660SH
A1
976AB
LB1
882RK
SK5
SAK7

Figure S3.

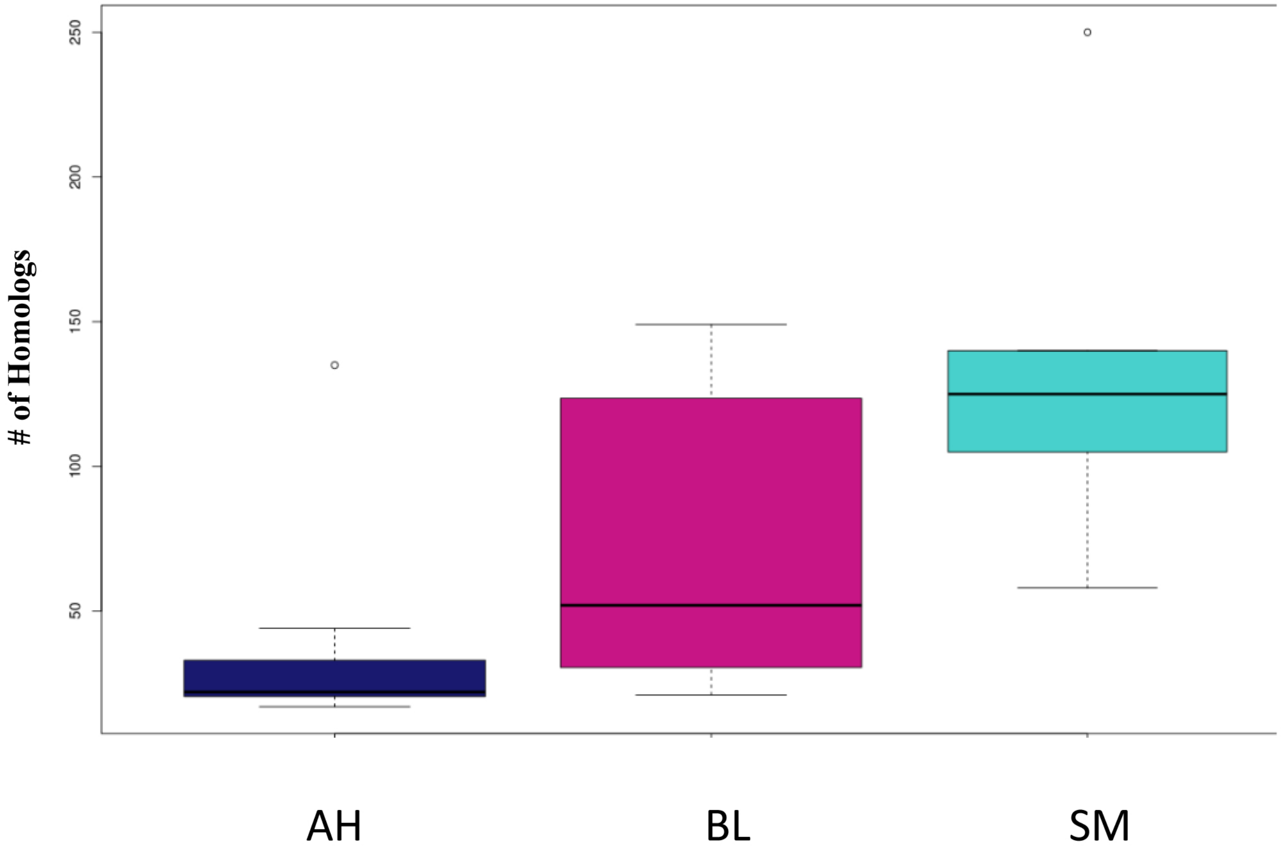

Figure S4.

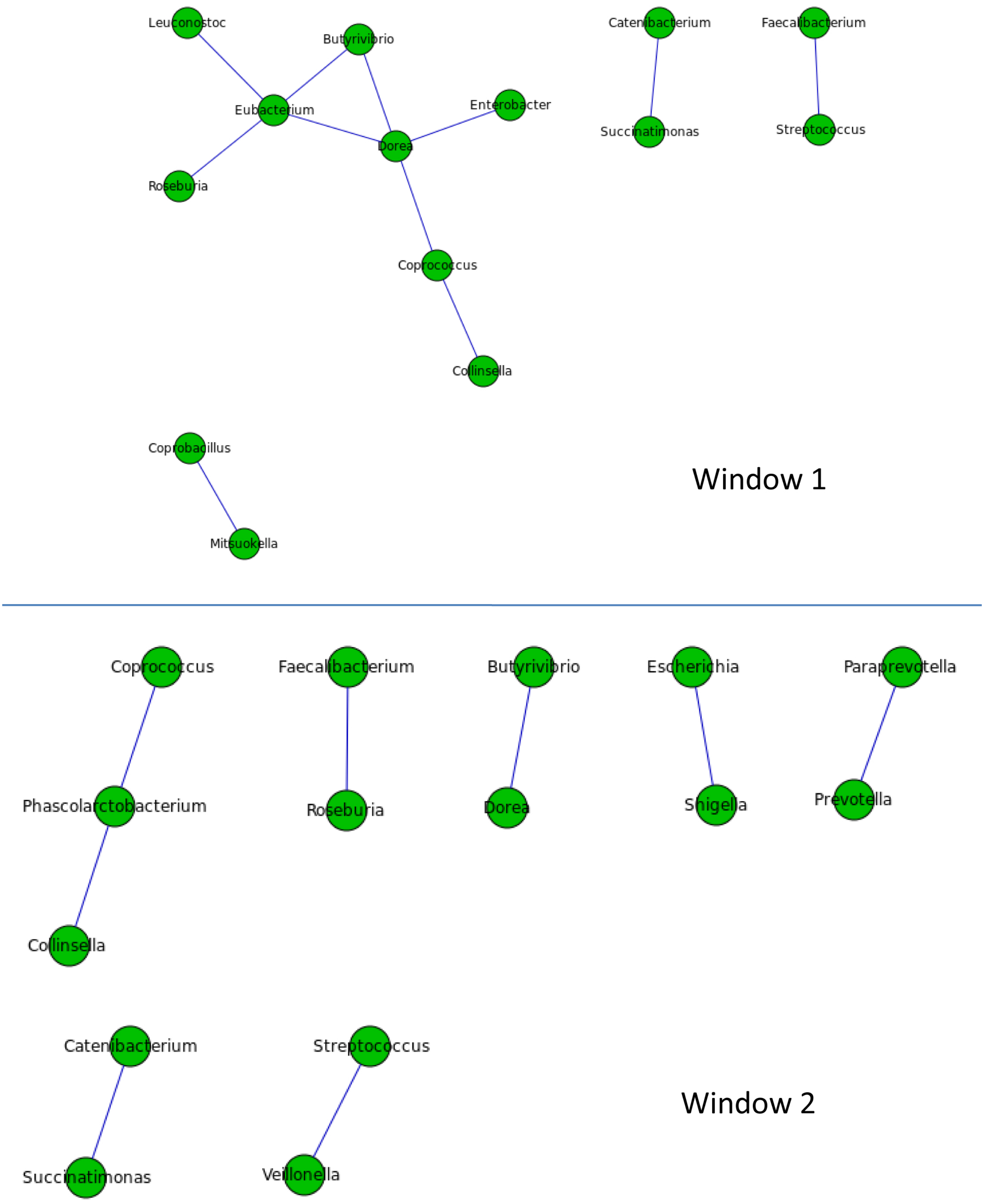
Leuconostoc
Butyrivibrio
Eubactetium
Enterobacter
Dorea
Roseburia
Coprococcus
Collinsella
Catenibacterium
Succinatimonas
Faecalibacterium
Streptococcus
Coprobacillus
Mitsuokella
Window 1
Coprococcus
Phascolarctobacterium
Collinsella
Faecalibacterium
Roseburia
Butyrivibrio
Dorea
Escherichia
Shigella
Paraprevotella
Prevotella
Catenibacterium
Succinatimonas
Streptococcus
Veillonella
Window 2

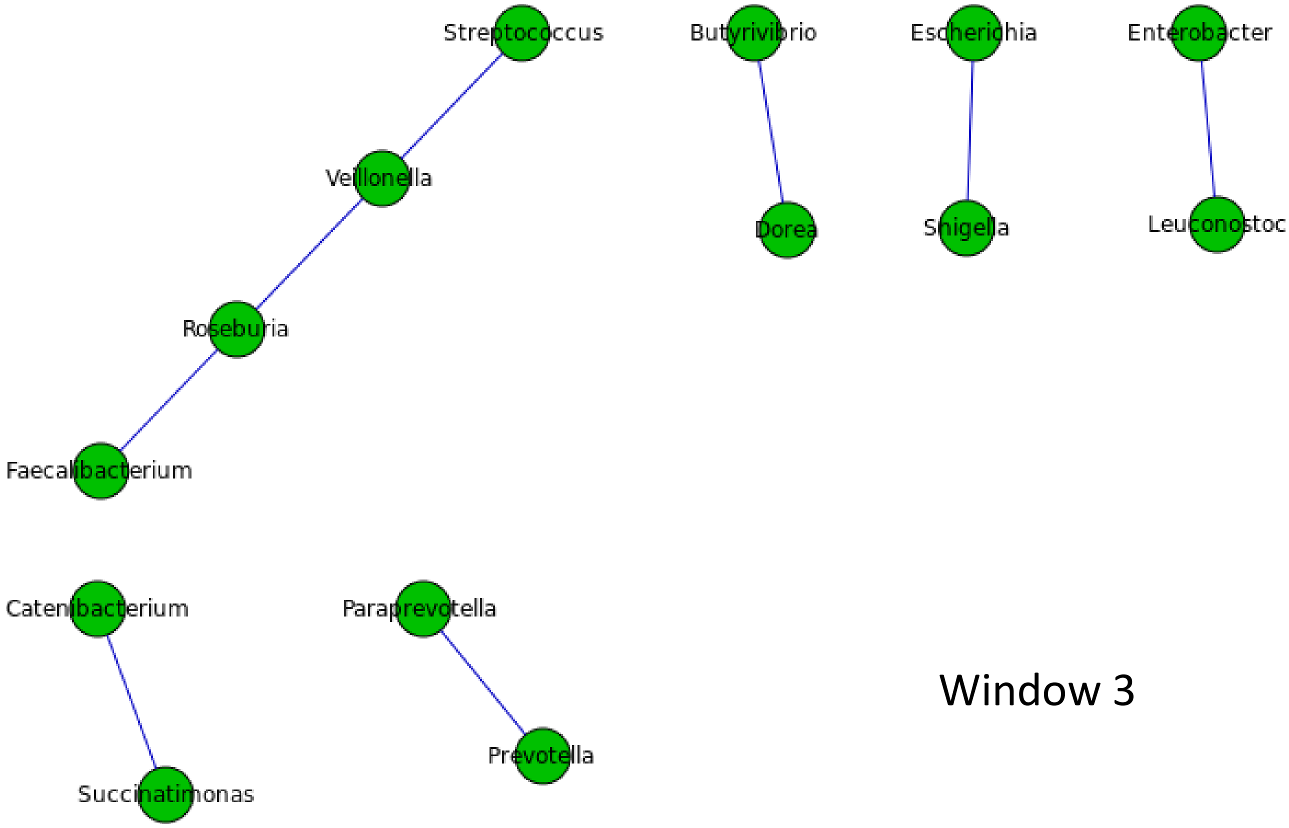
Streptococcus
Butyrivibrio
Escherichia
Enterobacter
Veillonella
Dorea
Shigella
Leuconostoc
Roseburia
Faecalibacterium
Catenibacterium
Paraprevotella
Window 3
Prevotella
Succinatimonas

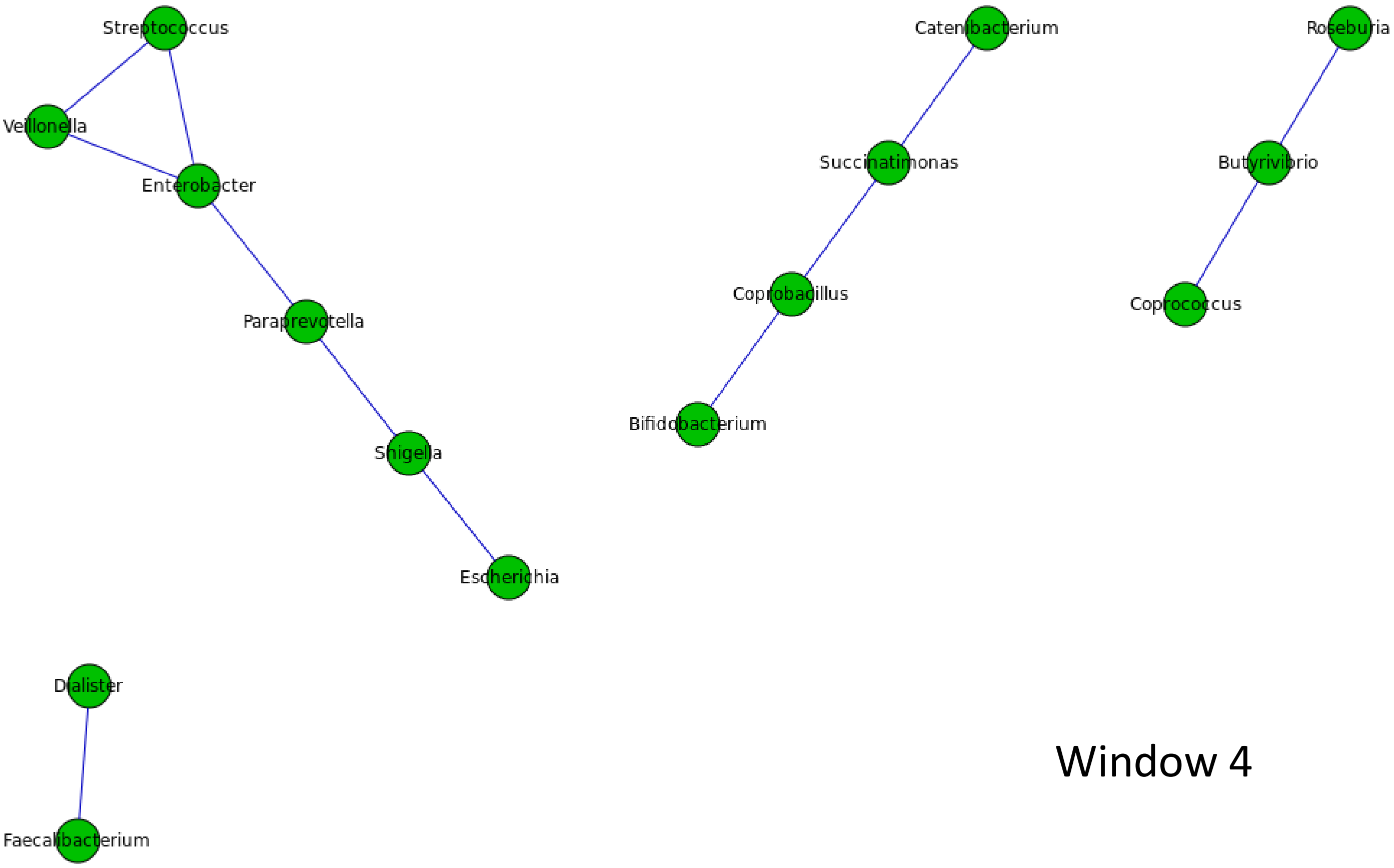
Streptococcus
Catenibacterium
Roseburia
Veillonella
Enterobacter
Succinatimonas
Butyrivibrio
Coprobacillus
Coprococcus
Paraprevotella
Bifidobacterium
Shigella
Escherichia
Dialister
Window 4
Faecalibacterium

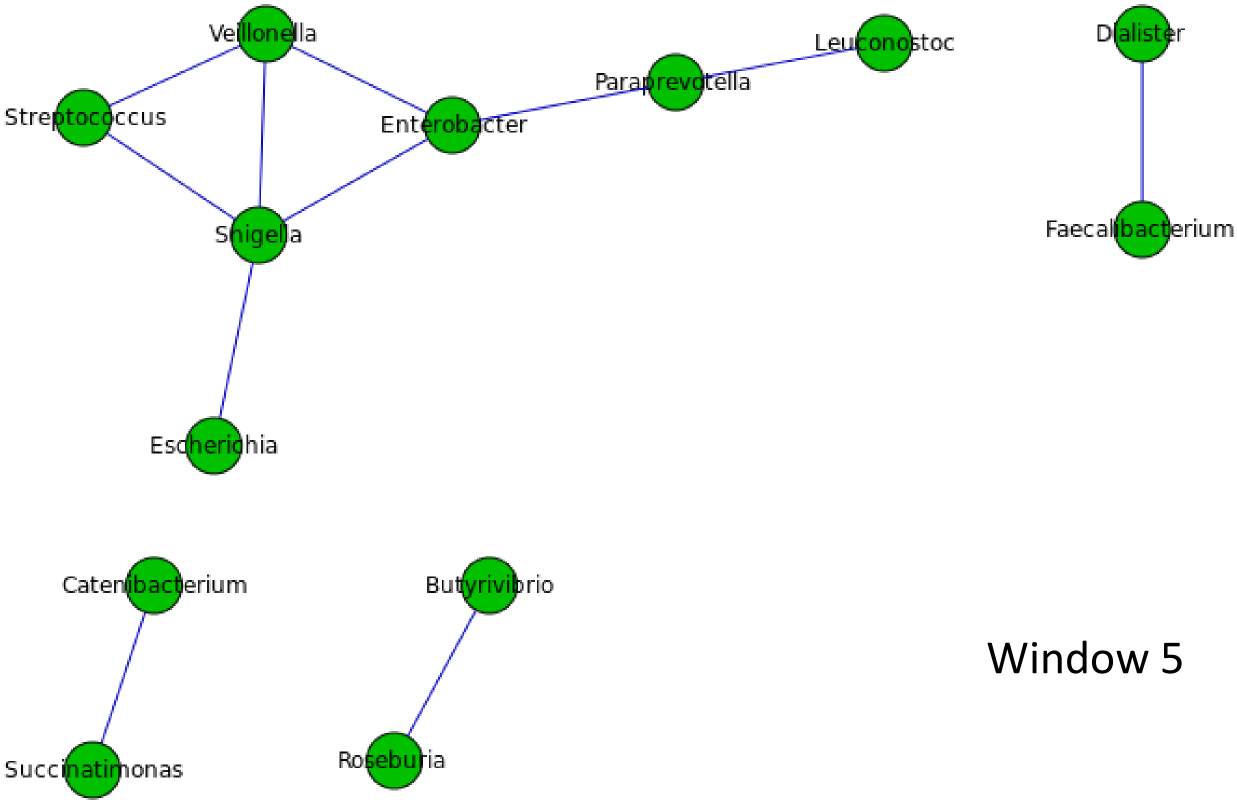

Window 5

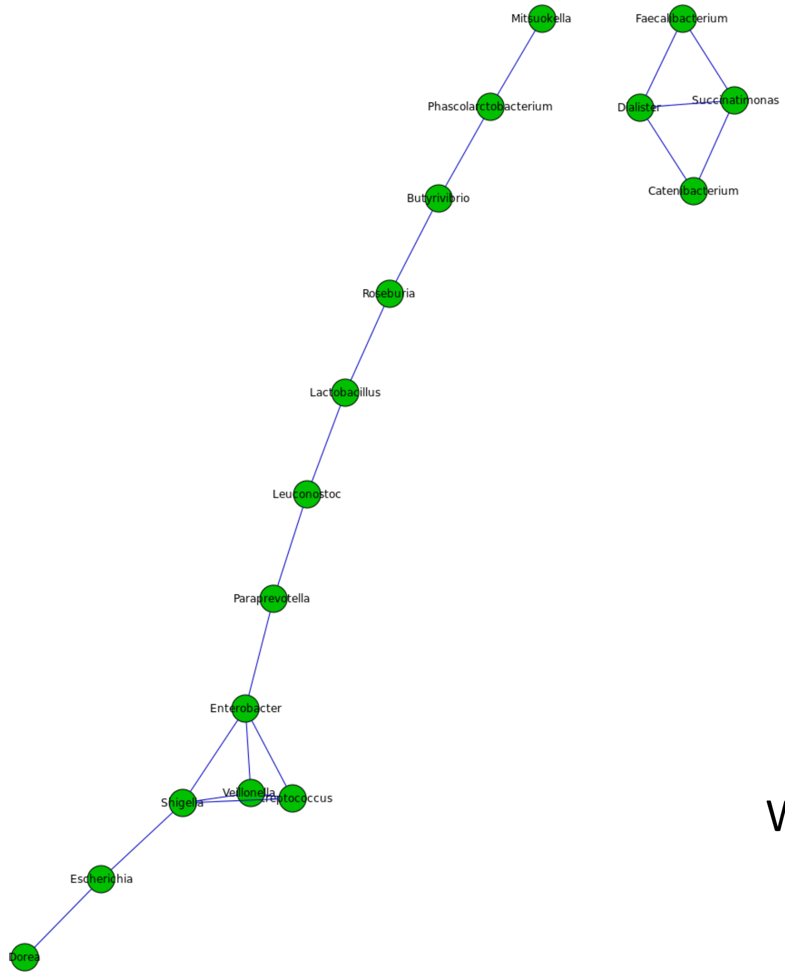

Window 6

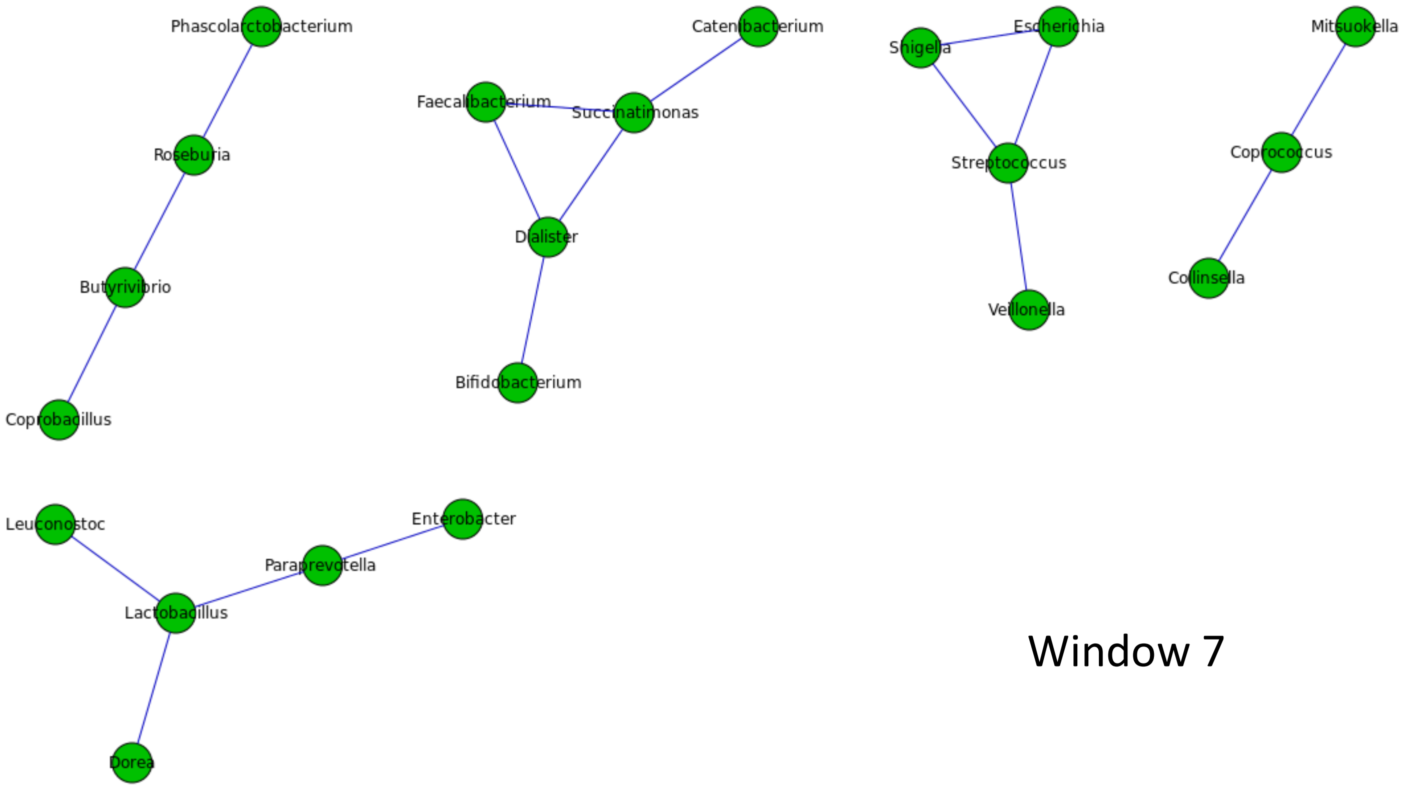

Window 7

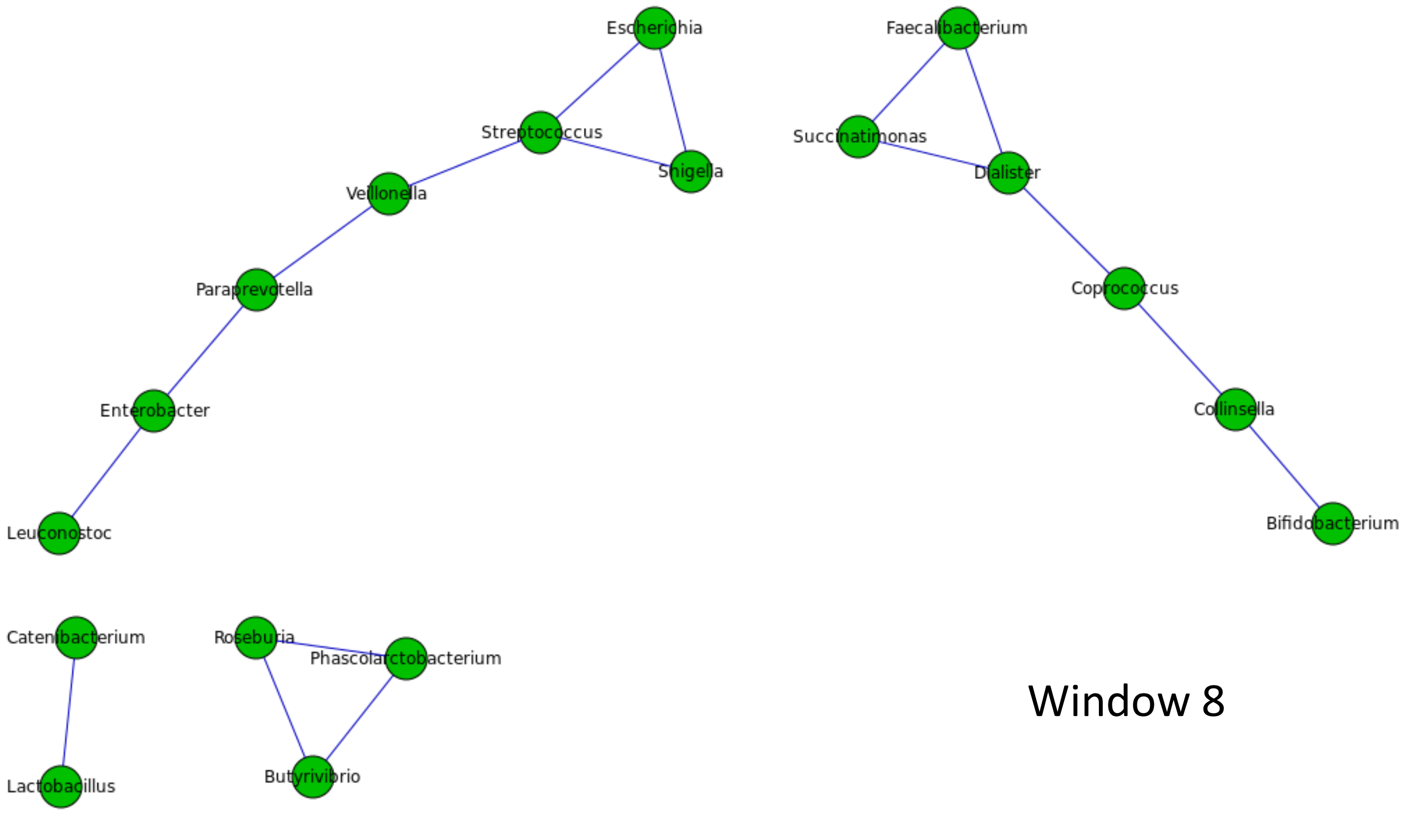

Window 8

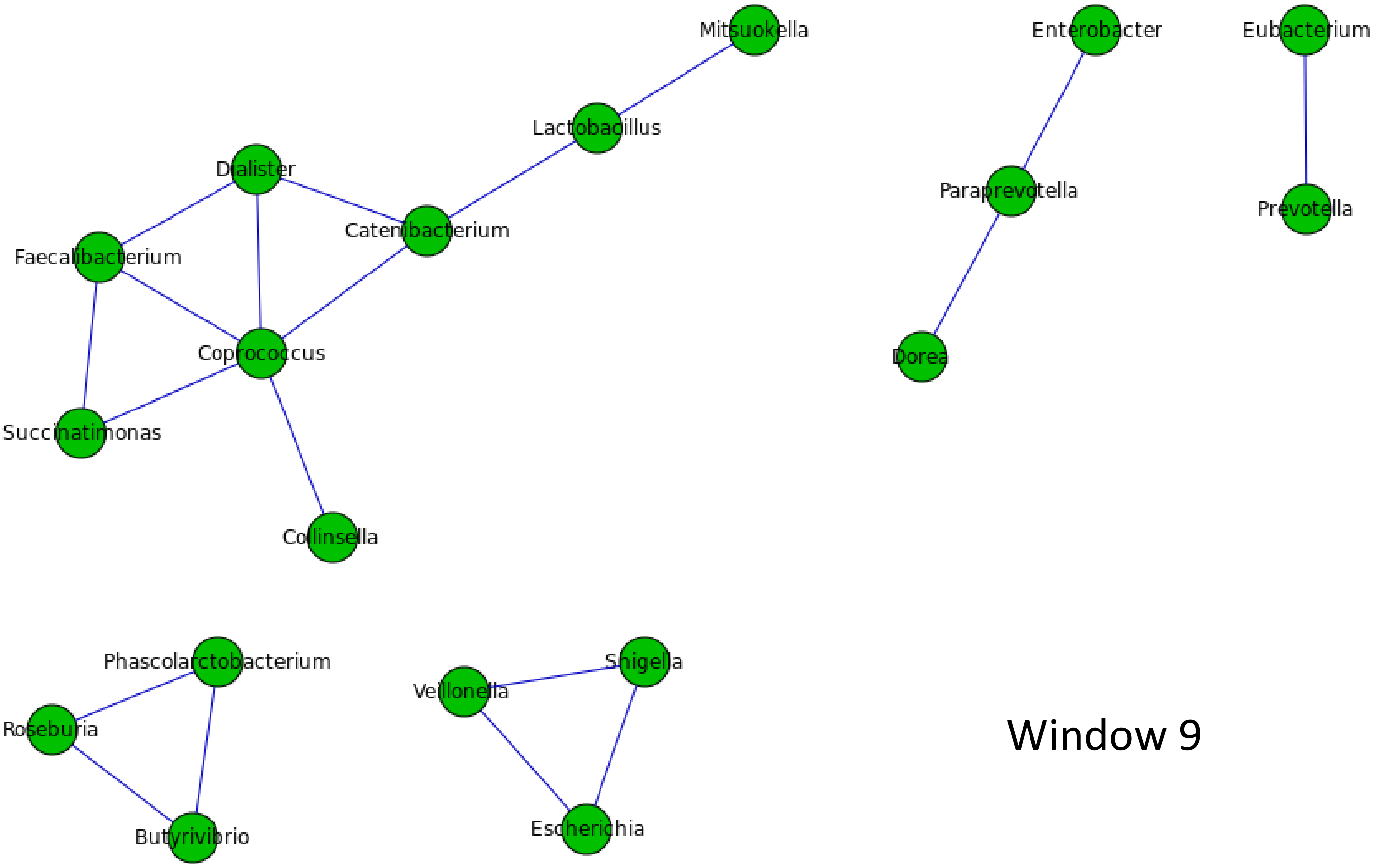

Window 9

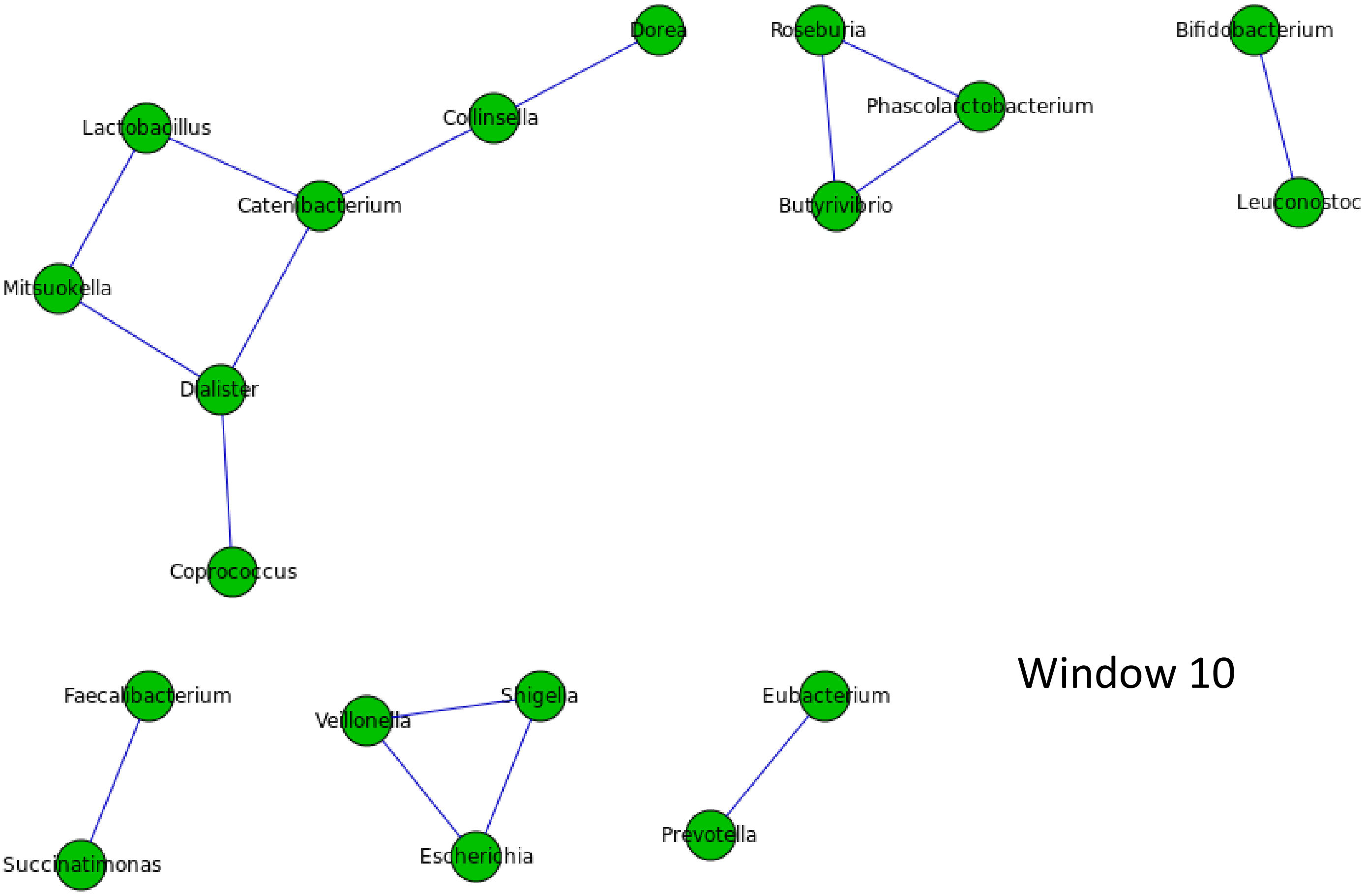

Window 10

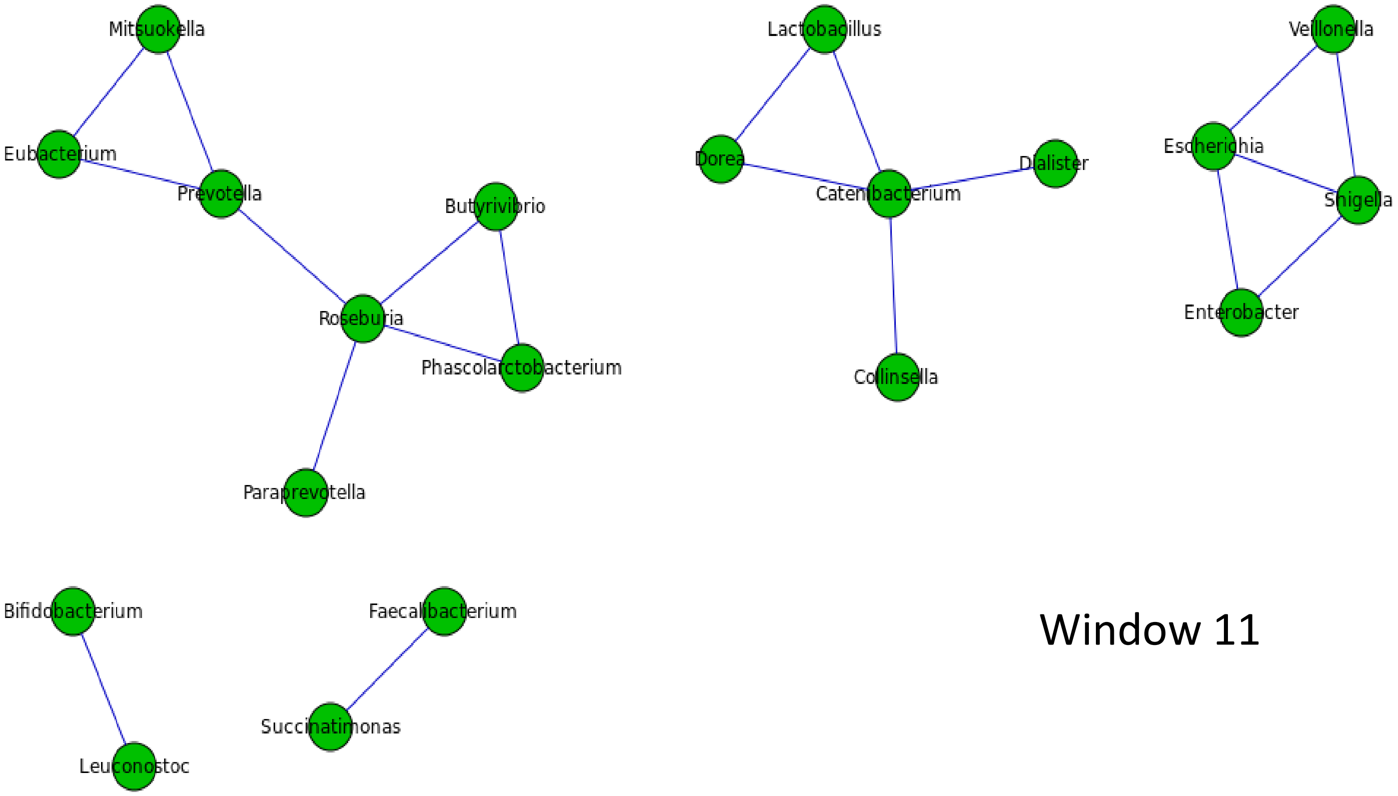

Window 11

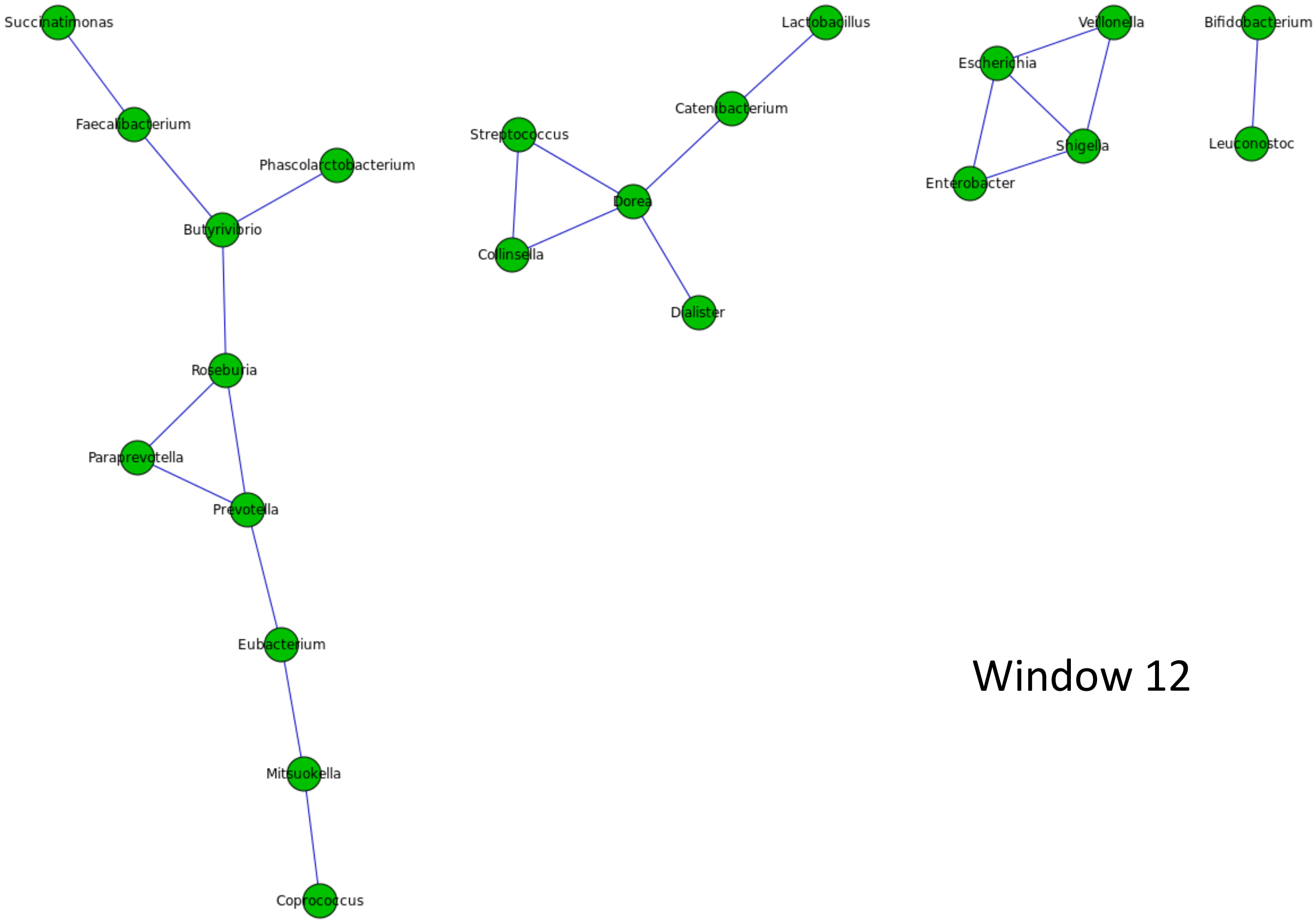

Window 12

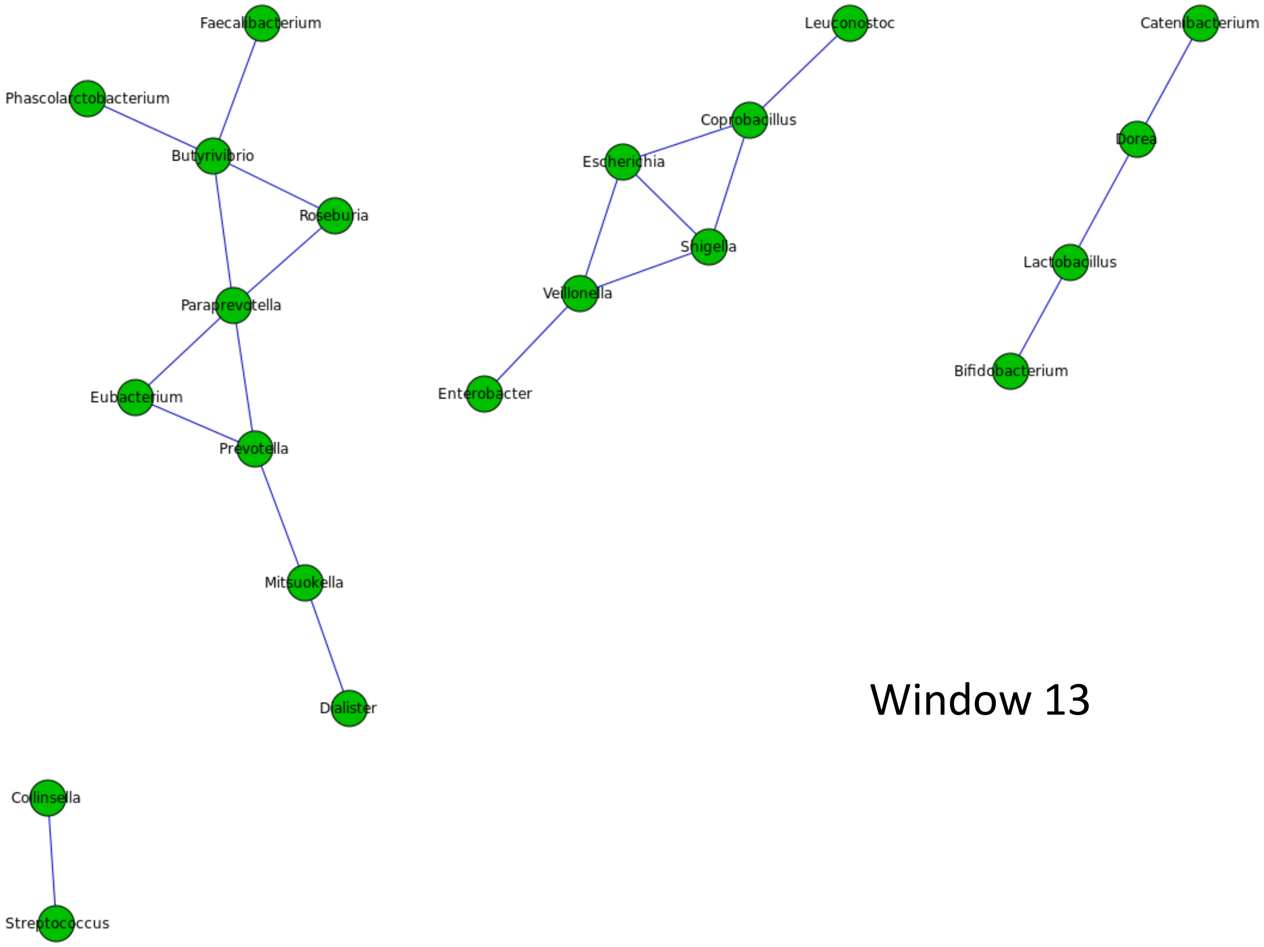
Faecalibacterium
Phascolarctobacterium
Butyrivibrio
Roseburia
Paraprevotella
Eubacterium
Prevotella
Mitsuokella
Dialister
Collinsella
Streptococcus
Leuconostoc
Coprobacillus
Escherichia
Shigella
Veillonella
Enterobacter
Catenibacterium
Dorea
Lactobacillus
Bifidobacterium
Window 13

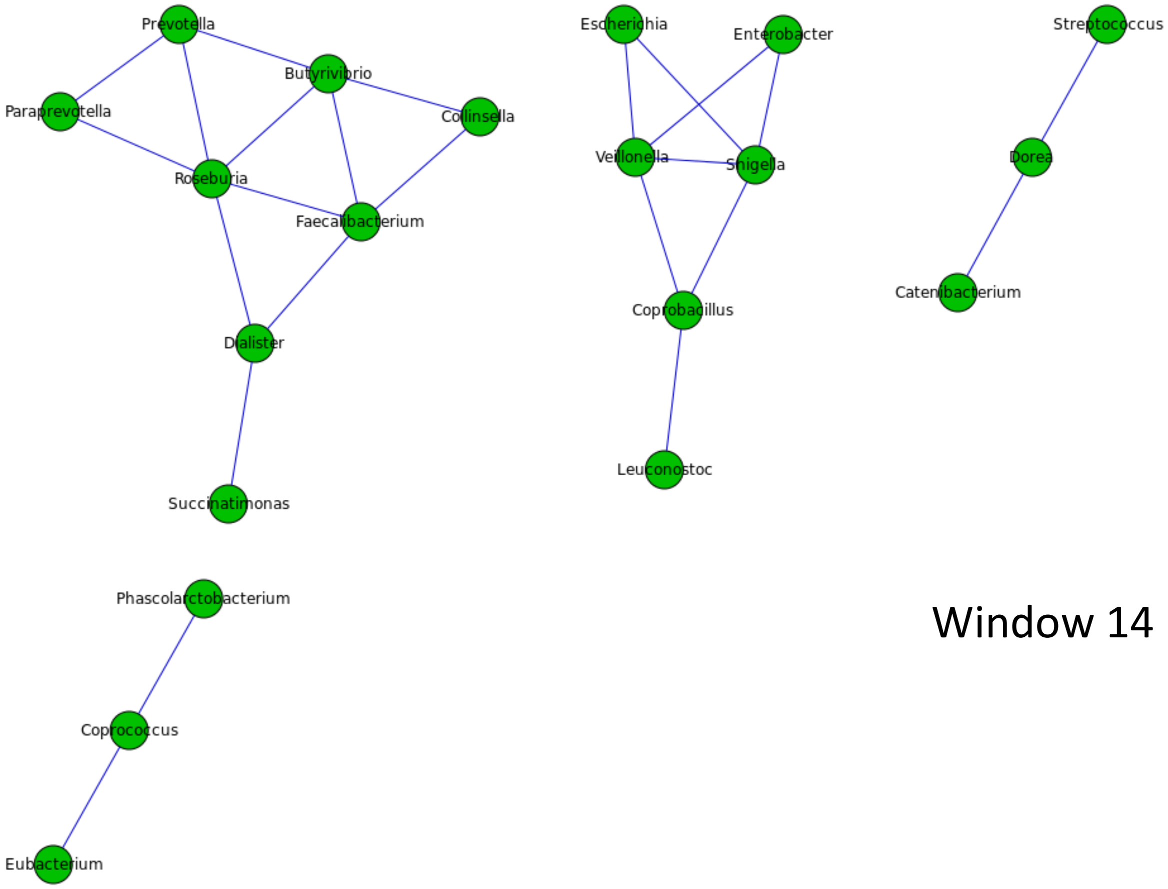
Prevotella
Butyrivibrio
Paraprevotella
Collinsella
Roseburia
Faecalibacterium
Dialister
Succinatimonas
Phascolarctobacterium
Coprococcus
Eubacterium
Escherichia
Enterobacter
Veillonella
Shigella
Coprobacillus
Leuconostoc
Streptococcus
Dorea
Catenibacterium
Window 14

Supplementary Table legends

Table S1.

Formulae used for the calculations of the various Z-scores for the 20 children.

Table S2.

Anthropometric data and sample data corresponding to the 20 children under study.

Table S3.

Percentages of sequences assigned to taxa at various levels across the 20 gut microbiomes.

Table S4.

Normalized abundances of various taxonomic groups at the (A) Genus, (B) Family, (C) Order, (D) Class and (E) Phylum.

Table S5.

Rank normalized abundances of various taxonomic groups at the (A) Genus, (B) Family, (C) Order, (D) Class and (E) Phylum.

Table S6.

Correlations of the different nutritional indices with the rank normalized abundances of various taxonomic groups at the levels of (A) Genus, (B) Family, (C) Order, (D) Class and (E) Phylum. Note that groups having a statistically significant positive or negative correlation with any one of

the nutritional indices have been highlighted in green and orange colors respectively, along with the corresponding Power values (both at $P<0.01$ and $P<0.05$), ReBOOT Z-values as well as the P-values computed using the ReBOOT method. The corresponding correlation values with a nutritional index have been indicated in *.

Table S7.

(A) Analysis of Variance (ANOVA) with multiple test correction (Benjamini-Hochberg FDR) and Post-hoc analysis of differences in pair of means using Tukey-Kramer's test.

(B) Kruskall-Wallis H-Test with Multiple test correction (Benjamini-Hochberg FDR) and Post-hoc analysis of differences in pair of means using Tukey-Kramer's test.

Table S8.

List of COGs (along with their correlation values) whose rank normalized abundances positively correlated with cumulative nutritional indices (Z-score) with a P-value < 0.05 (ReBoot Z-value < -1.97).

Table S9

List of COGs (along with their correlation values) whose rank normalized abundances negatively correlated with cumulative nutritional indices (Z-score) with a P-value < 0.05 (ReBOOT Z-value < -1.97).

Table S10

Group association of the CAZyme subfamilies based on similarities in their abundance patterns.

Table S11.

Pearson's Correlation Coefficients for each group against different nutritional indices. Score1 represents Height-for-Age; Score2 represents Weight-for-age; Score3 represents Weight-for-height and, overall represents the overall nutritional index, taking into account the previous 3 indexes. The boxes marked in green and red indicate significant correlation coefficients with P-value < 0.05 and P < 0.01 respectively.

Table S1.

Z-score1 (height, H for age, A)	$-3 + [\text{Height} - (-3\text{SD Value}_{H/A})] / [(-2\text{SD Value}_{H/A}) - (3\text{SD Value}_{H/A})]$
Z-score2 (weight, W for age, A)	$-3 + [\text{Height} - (-3\text{SD Value}_{W/A})] / [(-2\text{SD Value}_{W/A}) - (3\text{SD Value}_{W/A})]$
Z-score3 (weight, W for height, H)	$-3 + [\text{Height} - (-3\text{SD Value}_{W/H})] / [(-2\text{SD Value}_{W/H}) - (3\text{SD Value}_{W/H})]$

Table S2A.

Sample Name	Sample Details				Nutritional Indices (Z-Scores)				Metagenome Details		
	Age (in months)	Height (in cms)	Weight (in kgs)	Sex (M/F)	Z-score1 (Height for age)	Z-score2 (Weight for age)	Z-score3 (Weight for height)	Cumulative Z-score	No. of sequences	Average sequence length in base pairs (bp)	Metagenome Volume (in mega bps)
SAK7	60	90	9.4	F	-4.16	-4.32	-3.10	-11.58	1532351	389	596.1
SK5	48	82	8	F	-3.87	-4.12	-3.02	-11.01	1197527	421.7	505.0
882RK	60	92.6	9.8	F	-3.57	-4.11	-3.09	-10.77	964526	405.8	391.4
LB1	60	95	10	F	-3.02	-4	-3.33	-10.35	1423176	323.6	460.5
976AB	35	83.1	8.4	M	-2.95	-3.81	-3.1	-9.86	1376970	410.1	564.7
A1	48	90	10.9	M	-3.05	-3.05	-3.12	-9.22	1357415	321.8	436.8
1660SH	38	83.3	9.5	M	-3.36	-3.29	-2.1	-8.75	1294949	411.8	533.3
SH3	60	99	11	F	-2.11	-3.47	-3.08	-8.66	1321688	365.5	483.1
882AK	41	89.2	9.4	F	-2.10	-3.5	-2.92	-8.52	1780975	430.6	766.9
683RD	36	90	9.5	M	-1.29	-3.19	-3.27	-7.75	1339040	418.1	559.9
DB2	40	83	10.2	F	-3.54	-2.93	-1.1	-7.57	1529727	356.2	544.9
IS8	33	73.3	10	M	-5.27	-2.73	0.63	-7.37	1442484	373.4	538.6
199SUBD	30	87.3	8.9	F	-0.63	-3.08	-3.18	-6.90	1121174	421.6	472.7
U2	5	61.2	5	M	-2.37	-2.3	-1.33	-6.00	1869990	423.2	791.4
SOH4	36	90	11	M	-1.29	-2.25	-1.91	-5.45	1590763	304.9	485.0
976DB	19	76.9	8.5	F	-1.61	-2.09	1.6	-5.31	1211432	373.3	452.2
1660SK	57	98	14	F	-2.02	-1.74	-0.62	-4.38	1394515	384.4	536.1
683PD	48	98.8	14	M	-0.95	-1.42	-1.14	-3.51	1101390	392.4	432.2
AA6	33	89	12.5	M	-1.03	-1.07	-0.36	-2.46	1426324	422.4	602.5
199SD	48	101.3	12	F	2	-1.47	-2.71	-2.18	1858721	387.2	719.7

Table S2B.

Characteristics of Participants

	Mean (SD)	N (%/Absolute No.)
**Gender**		
Male		9 (45%)
Female		11 (55%)
Age Mean, Months (SD)	40.65 (15.06923)	
No. of family members Mean (SD)	6.20 (1.90843)	
Years of schooling Mean (SD)	2.80 (3.237983)	
Total annual income Median, Rs. (IQR)	31740 (8124.853)	Median - Rs.36000 (IQR -6000)
**Assets owned by family**		
House	0.90 (0.3077935)	18 (90%)
Television	0.25 (0.4442617)	6 (30%)
Cooler		0
Two wheeler		0
**Source of drinking water**		
Govt. piped supply		0
Govt. hand pump	0.90 (0.3077935)	18 (90%)
Family owned hand pump		0
Water tanker		0
Surface water	0.10 (0.3077935)	2 (10%)
Tube well		0
**Place of defecation**		
Pvt. toilet		0
Public toilet		0
Open field	1	20 (100%)
**Animal exposure**		
Cattle (Cows & Buffalo)	1.20 (1.321881)	10 (50%)
Others	2.45 (1.877148)	14 (70%)
**Consumption of foods (per child monthly consumption)**		
Cereals		
Wheat	0.30 (0.236198)	0.3 kg
Rice	2.10 (0.591163)	2.6 kg
Other cereals		
Pulses and legumes		
Dal	0.34 (0.1231174)	0.34 kg
Whole pulses		
Dairy products	300 (253.398)	300 ML
Fish	0.35 (0.2685242)	0.350 kg
Chicken	0.125 (0.0888523)	0.125 kg
Goat Meat	10 (30.77935)	10 gm
Egg	5.40 (4.357691)	5.4 (number)
Banana	2.30 (3.628832)	2.3 (number)
Coconut	0.15 (0.3284733)	0.153 (number)
Guava	0.75 (1.743409)	0.76 (number)
Vegetables		
Potato	1.40 (0.5758289)	1.4 kg
Green Leafy	0.18 (0.081757)	0.18 kg
Other Vegetables	1.60 (0.5026247)	1.6 kg

Table S3.

Metagenome	Percentage of sequences assigned at different taxonomic levels		
	Species	Genus or below	Phylum or below
SAK7	1.4	31.2	86.6
SK5	3.9	35.8	73.8
882RK	11	50.7	77.3
LB1	2.4	37.6	75
976AB	6.2	48.4	78.6
A1	1.4	43.2	67.8
1660SH	7.3	43	75.5
SH3	1.8	37.9	74.1
882AK	6	42.6	75.5
683RD	2.9	29.8	69.3
DB2	2.5	34.7	72.4
IS8	3.6	34.6	72.1
199SUBD	2	21.8	61.8
U2	2.7	38.4	70.4
SOH4	4.7	31.9	70.9
976DB	4.1	30.5	67.9
1660SK	1.7	22.6	63.2
683PD	12.4	49.1	77
199SD	5.1	36.1	72.1

Table S4.

(A)

GENUS	SAK7	SK5	882RK	LB1	976AB	A1	1660SH	SH3	882AK	683RD	DB2	IS8	199SUBD	U2	SOH4	976DB	1660SK	683PD	AA6	199SD
Dorea	0.11	0.09	0.18	0.11	0.21	0.00	0.15	0.09	0.21	0.10	0.15	0.16	0.11	0.16	0.07	0.08	0.47	0.11	0.15	0.06
Leuconostoc	0.00	0.00	0.12	0.00	0.02	0.03	0.00	0.00	0.00	0.00	0.00	0.01	0.00	0.01	0.00	0.00	0.01	0.01	0.00	0.00
Dialister	0.04	0.11	0.03	0.07	0.08	0.00	0.09	0.01	0.10	0.22	0.04	0.13	0.11	0.03	0.12	0.00	0.16	0.00	0.18	0.05
Succinatimonas	0.00	0.08	0.00	0.01	0.02	0.00	0.00	0.02	0.03	0.16	0.00	0.03	0.02	0.00	0.02	0.32	0.12	0.00	0.12	0.00
Collinsella	0.04	0.08	0.07	0.12	0.52	0.00	0.05	0.01	0.07	0.13	0.11	0.05	0.15	0.22	0.06	0.17	0.11	0.06	0.06	0.02
Phascolarctobacterium	0.00	0.02	0.05	0.13	0.05	0.00	0.04	0.41	0.00	0.01	0.00	0.01	0.28	0.29	0.01	0.72	0.31	0.00	0.21	0.34
Veillonella	0.04	1.07	1.75	0.13	0.07	6.13	0.09	0.48	0.30	0.01	0.95	0.35	0.04	0.04	0.08	0.02	0.11	0.25	0.01	0.02
Roseburia	0.13	0.27	0.10	0.32	0.14	0.00	0.25	0.72	0.22	0.12	0.10	0.71	0.26	0.50	0.35	0.22	1.06	1.72	1.04	0.13
Catenibacterium	0.11	0.08	1.77	0.04	1.81	0.00	0.15	0.04	0.12	0.34	0.04	0.34	0.02	0.00	0.09	0.10	0.35	0.07	0.12	0.07
Prevotella	24.88	27.81	13.49	29.18	22.21	0.08	35.65	26.53	25.69	23.08	26.02	23.51	13.03	28.76	24.84	23.67	10.51	34.75	13.67	30.01
Faecalibacterium	0.65	0.87	0.49	0.88	0.97	0.00	0.58	0.95	0.89	1.56	0.79	1.22	1.19	0.46	0.78	0.50	1.82	2.20	1.36	1.38
Streptococcus	0.24	0.75	5.13	1.72	11.26	0.29	0.11	0.35	1.78	0.02	0.50	0.33	0.03	0.06	0.14	0.08	0.15	0.18	0.04	0.16
Shigella	0.02	0.04	0.11	0.03	0.02	1.02	0.01	0.09	0.22	0.00	0.05	0.02	0.00	0.00	0.02	0.00	0.00	0.00	0.12	0.00
Escherichia	0.71	1.27	5.51	1.19	0.56	26.55	0.21	4.79	8.40	0.15	1.49	0.75	0.08	0.13	0.06	0.01	0.03	0.11	2.57	0.03
Coprococcus	0.05	0.09	0.10	0.17	0.07	0.00	0.07	0.06	0.14	0.17	0.08	0.14	0.28	0.17	0.11	0.16	0.39	0.07	0.14	0.05
Butyrivibrio	0.01	0.02	0.01	0.03	0.03	0.00	0.02	0.06	0.01	0.01	0.01	0.72	0.08	0.03	0.02	0.02	1.39	0.03	0.12	0.02
Eubacterium	0.49	0.44	0.72	0.91	0.28	0.01	2.22	0.81	0.96	0.30	0.92	0.59	1.69	1.44	0.86	0.73	3.66	2.57	1.21	0.30
Bifidobacterium	0.02	0.04	0.61	0.00	3.18	2.63	0.07	0.00	0.08	0.59	0.06	0.05	0.12	0.01	0.22	0.40	0.08	0.33	0.06	0.00
Coprobacillus	0.00	0.03	0.16	0.01	0.05	1.38	0.00	0.01	0.00	0.01	0.01	0.01	0.02	0.01	0.02	0.04	0.02	0.00	0.01	0.01
Paraprevotella	0.32	0.26	0.16	0.35	0.25	0.00	0.27	0.29	0.29	0.18	0.31	0.34	0.16	0.34	0.25	0.19	0.12	0.44	0.23	0.22
Mitsuokella	0.16	0.07	0.03	0.03	0.02	0.00	0.33	0.00	0.03	0.01	0.00	0.01	0.24	0.14	1.14	0.11	0.25	0.01	0.05	0.07
Enterobacter	0.00	0.99	0.01	0.01	0.01	0.01	0.00	0.01	0.01	0.00	0.02	0.14	0.00	0.01	0.00	0.00	0.00	0.00	0.04	0.00
Lactobacillus	0.03	0.00	17.13	0.01	4.24	0.04	0.23	0.00	0.11	0.09	0.02	0.40	0.02	0.17	0.18	0.25	0.03	0.04	0.02	0.01

(B)

FAMILY	SAK7	SK5	882RK	LB1	976AB	A1	1660SH	SH3	882AK	683RD	DB2	IS8	199SUBD	U2	SOH4	976DB	1660SK	683PD	AA6	199SD
Veillonellaceae	0.26	1.27	1.83	0.24	0.18	6.14	0.54	0.50	0.45	0.25	1.00	0.50	0.41	0.22	1.48	0.19	0.55	0.29	0.26	0.14
Porphyromonadaceae	0.19	0.28	0.12	0.29	0.23	0.01	0.29	0.31	0.31	0.53	0.34	0.22	0.25	0.40	0.31	0.24	0.19	0.39	0.26	0.27
Desulfovibrionaceae	0.03	0.01	0.01	0.01	0.00	0.06	0.01	0.03	0.00	0.07	0.01	0.01	0.05	0.00	0.05	0.02	0.07	0.04	0.25	0.00
Bacteroidaceae	2.44	2.20	0.60	2.26	1.32	0.08	2.21	2.36	1.47	2.41	2.91	1.85	1.35	4.28	2.23	1.55	1.08	5.22	1.91	1.98
Lactobacillaceae	0.03	0.00	17.13	0.01	4.24	0.04	0.23	0.00	0.11	0.09	0.02	0.40	0.02	0.17	0.18	0.25	0.03	0.04	0.02	0.01
Streptococcaceae	0.24	0.75	5.13	1.72	11.28	0.29	0.11	0.35	1.79	0.02	0.50	0.33	0.03	0.09	0.14	0.08	0.15	0.19	0.04	0.16
Enterobacteriaceae	0.76	3.17	5.70	1.40	0.66	28.02	0.25	4.94	8.80	0.19	1.95	1.10	0.10	0.16	0.12	0.03	0.07	0.13	2.82	0.04
Bifidobacteriaceae	0.02	0.04	0.61	0.00	3.18	2.63	0.07	0.00	0.08	0.59	0.06	0.05	0.12	0.01	0.22	0.40	0.08	0.33	0.06	0.00
Coriobacteriaceae	0.05	0.09	0.09	0.13	0.54	0.20	0.05	0.02	0.08	0.14	0.12	0.06	0.18	0.23	0.07	0.19	0.14	0.07	0.08	0.02
Lachnospiraceae	0.34	0.52	0.48	0.69	0.64	0.02	0.54	0.96	0.64	0.46	0.37	1.84	0.84	0.93	0.61	0.51	3.49	2.14	1.58	0.30
Pasteurellaceae	0.07	0.15	0.37	0.01	0.20	0.01	0.07	0.09	0.01	0.06	0.08	0.10	0.05	0.07	0.04	0.04	0.09	0.06	0.02	0.13
Prevotellaceae	25.21	28.06	13.64	29.53	22.46	0.08	35.92	26.82	25.98	23.26	26.33	23.85	13.19	29.10	25.09	23.86	10.63	35.19	13.90	30.24
Clostridiaceae	0.07	0.15	2.42	0.15	0.26	0.04	0.17	0.17	0.16	0.19	0.14	0.31	0.52	0.13	0.20	0.13	0.77	0.28	0.39	0.14
Succinivibrionaceae	0.00	0.08	0.00	0.01	0.02	0.00	0.00	0.02	0.03	0.16	0.00	0.03	0.02	0.00	0.02	0.32	0.12	0.00	0.12	0.00
Acidaminococcaceae	0.03	0.05	0.07	0.17	0.07	0.12	0.06	0.43	0.22	0.03	0.02	0.06	0.30	0.31	0.06	0.74	0.34	0.01	0.25	0.35
Rikenellaceae	0.01	0.02	0.01	0.03	0.02	0.10	0.02	0.01	0.01	0.03	0.03	0.07	0.03	0.01	0.10	0.05	0.03	0.01	0.11	0.01
Erysipelotrichaceae	0.22	0.20	2.01	0.18	2.05	1.38	0.20	0.10	0.19	0.70	0.13	0.64	0.23	0.30	0.20	0.32	0.59	0.16	0.25	0.21
Eubacteriaceae	0.49	0.44	0.72	0.91	0.28	0.02	2.22	0.81	0.96	0.30	0.92	0.60	1.70	1.44	0.86	0.73	3.67	2.57	1.21	0.30
Ruminococcaceae	0.73	1.07	0.78	1.08	1.38	0.00	0.80	1.12	1.16	1.71	0.88	1.90	1.74	0.71	0.94	0.69	2.65	2.76	1.70	1.56
Sutterellaceae	0.12	0.03	0.06	0.01	0.01	0.00	0.12	0.01	0.01	0.03	0.04	0.01	0.00	0.02	0.02	0.05	0.02	0.00	0.03	0.00
Leuconostocaceae	0.00	0.01	0.24	0.00	0.03	0.03	0.00	0.00	0.00	0.00	0.00	0.02	0.00	0.01	0.01	0.00	0.01	0.01	0.00	0.00

(C)

ORDER	SAK7	SK5	882RK	LB1	976AB	A1	1660SH	SH3	882AK	683RD	DB2	IS8	199SUBD	U2	SOH4	976DB	1660SK	683PD	AA6	199SD
Aeromonadales	0.00	0.08	0.00	0.01	0.02	0.00	0.00	0.02	0.04	0.16	0.00	0.03	0.02	0.00	0.02	0.33	0.12	0.00	0.12	0.00
Erysipelotrichales	0.22	0.20	2.01	0.18	2.05	1.38	0.20	0.10	0.19	0.70	0.13	0.64	0.23	0.30	0.20	0.32	0.59	0.16	0.25	0.21
Desulfovibrionales	0.03	0.01	0.01	0.01	0.00	0.06	0.01	0.03	0.00	0.07	0.01	0.01	0.05	0.00	0.05	0.02	0.07	0.04	0.25	0.00
Pasteurellales	0.07	0.15	0.37	0.01	0.20	0.01	0.07	0.09	0.01	0.06	0.08	0.10	0.05	0.07	0.04	0.04	0.09	0.06	0.02	0.13
Selenomonadales	0.29	1.31	1.90	0.41	0.24	6.26	0.59	0.93	0.68	0.28	1.01	0.57	0.71	0.53	1.54	0.93	0.89	0.30	0.51	0.49
Lactobacillales	0.27	0.79	22.57	1.76	15.59	0.82	0.35	0.37	1.97	0.11	0.53	0.76	0.06	0.28	0.33	0.34	0.21	0.24	0.07	0.17
Enterobacteriales	0.76	3.17	5.70	1.40	0.66	28.02	0.25	4.94	8.80	0.19	1.95	1.10	0.10	0.16	0.12	0.03	0.07	0.13	2.82	0.04
Bifidobacteriales	0.02	0.04	0.61	0.00	3.18	2.63	0.07	0.00	0.08	0.59	0.06	0.05	0.12	0.01	0.22	0.40	0.08	0.33	0.06	0.00
Clostridiales	1.66	2.24	4.51	2.88	2.75	0.15	3.76	3.11	2.96	2.73	2.38	4.78	5.16	3.24	2.82	2.11	10.85	7.82	5.09	2.33
Spirochaetales	0.00	0.00	0.01	0.00	1.09	0.02	0.00	0.00	0.00	0.08	0.00	0.57	0.16	0.00	0.00	0.00	0.25	0.00	0.00	0.15
Coriobacteriales	0.05	0.09	0.09	0.13	0.54	0.20	0.05	0.02	0.08	0.14	0.12	0.06	0.18	0.23	0.07	0.19	0.14	0.07	0.08	0.02
Bacteroidales	27.84	30.57	14.37	32.12	24.02	0.27	38.44	29.50	27.77	26.23	29.63	26.00	14.82	33.78	27.73	25.70	11.92	40.82	16.19	32.50
Burkholderiales	0.12	0.04	0.07	0.03	0.01	0.02	0.13	0.02	0.01	0.04	0.05	0.03	0.03	0.02	0.04	0.06	0.04	0.00	0.05	0.01

(D)

CLASS	SAK7	SK5	882RK	LB1	976AB	A1	1660SH	SH3	882AK	683RD	DB2	IS8	199SUBD	U2	SOH4	976DB	1660SK	683PD	AA6	199SD
Alphaproteobacteria	0.00	0.01	0.01	0.02	0.01	0.02	0.01	0.01	0.01	0.01	0.01	0.03	0.02	0.01	0.02	0.01	0.03	0.01	0.02	0.01
Deltaproteobacteria	0.03	0.02	0.01	0.02	0.01	0.07	0.02	0.03	0.02	0.10	0.02	0.03	0.07	0.01	0.06	0.02	0.09	0.04	0.26	0.01
Gammaproteobacteria	0.83	3.41	6.09	1.45	0.89	28.09	0.34	5.06	8.86	0.44	2.05	1.28	0.19	0.24	0.22	0.44	0.32	0.20	2.99	0.19
Erysipelotrichi	0.22	0.20	2.01	0.18	2.05	1.38	0.20	0.10	0.19	0.70	0.13	0.64	0.23	0.30	0.20	0.32	0.59	0.16	0.25	0.21
Spirochaetia	0.00	0.00	0.01	0.00	1.09	0.02	0.00	0.00	0.00	0.08	0.00	0.57	0.16	0.00	0.00	0.00	0.25	0.00	0.00	0.15
Bacteroidia	27.84	30.57	14.37	32.12	24.02	0.27	38.44	29.50	27.77	26.23	29.63	26.00	14.82	33.78	27.73	25.70	11.92	40.82	16.19	32.50
Negativicutes	0.29	1.31	1.90	0.41	0.24	6.26	0.59	0.93	0.68	0.28	1.01	0.57	0.71	0.53	1.54	0.93	0.89	0.30	0.51	0.49
Clostridia	1.66	2.24	4.51	2.88	2.75	0.17	3.76	3.11	2.96	2.73	2.38	4.78	5.16	3.24	2.82	2.11	10.85	7.82	5.09	2.33
Actinobacteria (class)	0.07	0.15	0.72	0.15	3.73	2.88	0.13	0.04	0.17	0.75	0.19	0.13	0.33	0.24	0.31	0.59	0.25	0.41	0.16	0.03
Bacilli	0.27	0.80	22.60	1.77	15.60	0.87	0.36	0.38	1.98	0.12	0.54	0.78	0.07	0.29	0.35	0.35	0.22	0.25	0.08	0.18
Betaproteobacteria	0.13	0.08	0.08	0.04	0.04	0.06	0.13	0.08	0.01	0.05	0.08	0.04	0.04	0.02	0.05	0.06	0.06	0.01	0.07	0.01

(E)

PHYLUM	SAK7	SK5	882RK	LB1	976AB	A1	1660SH	SH3	882AK	683RD	DB2	IS8	199SUBD	U2	SOH4	976DB	1660SK	683PD	AA6	199SD
Bacteroidetes	71.73	56.56	23.22	60.06	42.33	1.63	63.93	56.96	52.41	52.59	59.09	48.61	34.80	59.50	52.53	48.16	28.56	61.20	36.82	59.48
Fusobacteria	0.53	0.42	1.40	0.28	0.49	9.06	0.24	0.28	0.22	0.27	0.34	0.37	0.42	0.26	0.45	0.66	0.47	0.23	0.33	0.36
Euryarchaeota	0.27	0.16	0.18	0.19	0.14	0.71	0.17	0.16	0.13	0.16	0.19	0.19	0.38	0.11	0.24	0.22	0.28	0.14	0.20	0.21
Actinobacteria	0.48	0.42	0.96	0.56	4.22	3.36	0.37	0.30	0.41	1.19	0.59	0.58	1.24	0.43	0.98	0.86	1.03	0.69	0.78	0.29
Proteobacteria	3.32	6.50	7.77	3.43	2.88	33.37	1.93	7.09	10.85	4.20	3.90	4.35	3.03	1.58	3.62	7.24	4.94	1.50	7.00	1.89
Spirochaetes	0.16	0.08	0.12	0.10	2.98	0.36	0.08	0.10	0.08	0.25	0.10	1.16	1.08	0.09	0.14	0.17	0.77	0.10	0.11	0.91
Synergistetes	0.02	0.02	0.01	0.03	0.01	0.01	0.01	0.02	0.02	0.03	0.02	0.04	0.13	0.01	0.04	0.01	0.08	0.01	0.08	0.02
Firmicutes	8.63	9.86	43.85	10.59	25.74	17.65	8.97	9.37	10.94	10.74	8.37	16.23	19.90	8.10	13.18	9.77	27.47	13.24	18.13	8.30

Table S5.

(A)

GENUS	SAK7	SK5	882RK	LB1	976AB	A1	1660SH	SH3	882AK	683RD	DB2	IS8	199SUBD	U2	SOH4	976DB	1660SK	683PD	AA6	199SD
Dorea	0.37	0.26	0.84	0.47	0.95	0.00	0.58	0.21	0.89	0.32	0.63	0.79	0.42	0.74	0.11	0.16	1.00	0.53	0.68	0.05
Leuconostoc	0.00	0.58	1.00	0.37	0.89	0.95	0.11	0.32	0.63	0.47	0.26	0.79	0.05	0.84	0.53	0.21	0.68	0.74	0.42	0.16
Dialister	0.32	0.68	0.26	0.47	0.53	0.00	0.58	0.16	0.63	1.00	0.37	0.84	0.74	0.21	0.79	0.05	0.89	0.11	0.95	0.42
Succinatimonas	0.11	0.79	0.26	0.42	0.58	0.21	0.16	0.53	0.74	0.95	0.32	0.68	0.63	0.00	0.47	1.00	0.89	0.05	0.84	0.37
Collinsella	0.16	0.58	0.47	0.74	1.00	0.00	0.26	0.05	0.53	0.79	0.63	0.21	0.84	0.95	0.32	0.89	0.68	0.37	0.42	0.11
Phascolarctobacterium	0.05	0.42	0.58	0.63	0.53	0.00	0.47	0.95	0.21	0.26	0.11	0.32	0.74	0.79	0.37	1.00	0.84	0.16	0.68	0.89
Veillonella	0.32	0.89	0.95	0.58	0.37	1.00	0.47	0.79	0.68	0.05	0.84	0.74	0.21	0.26	0.42	0.16	0.53	0.63	0.00	0.11
Roseburia	0.21	0.58	0.11	0.63	0.32	0.00	0.47	0.84	0.42	0.16	0.05	0.79	0.53	0.74	0.68	0.37	0.95	1.00	0.89	0.26
Catenibacterium	0.58	0.42	0.95	0.21	1.00	0.00	0.74	0.16	0.63	0.79	0.26	0.84	0.11	0.05	0.47	0.53	0.89	0.32	0.68	0.37
Prevotella	0.53	0.74	0.16	0.84	0.26	0.00	1.00	0.68	0.58	0.32	0.63	0.37	0.11	0.79	0.47	0.42	0.05	0.95	0.21	0.89
Faecalibacterium	0.26	0.42	0.11	0.47	0.63	0.00	0.21	0.58	0.53	0.89	0.37	0.74	0.68	0.05	0.32	0.16	0.95	1.00	0.79	0.84
Streptococcus	0.53	0.79	0.95	0.84	1.00	0.58	0.26	0.68	0.89	0.00	0.74	0.63	0.05	0.16	0.32	0.21	0.37	0.47	0.11	0.42
Shigella	0.53	0.68	0.84	0.63	0.58	1.00	0.37	0.79	0.95	0.21	0.74	0.47	0.11	0.26	0.42	0.05	0.00	0.16	0.89	0.32
Escherichia	0.53	0.68	0.89	0.63	0.47	1.00	0.42	0.84	0.95	0.37	0.74	0.58	0.21	0.32	0.16	0.00	0.05	0.26	0.79	0.11
Coprococcus	0.05	0.42	0.47	0.84	0.21	0.00	0.32	0.16	0.58	0.79	0.37	0.63	0.95	0.89	0.53	0.74	1.00	0.26	0.68	0.11
Butyrivibrio	0.11	0.37	0.05	0.74	0.58	0.00	0.32	0.79	0.21	0.26	0.16	0.95	0.84	0.68	0.42	0.53	1.00	0.63	0.89	0.47
Eubacterium	0.26	0.21	0.37	0.58	0.05	0.00	0.89	0.47	0.68	0.16	0.63	0.32	0.84	0.79	0.53	0.42	1.00	0.95	0.74	0.11
Bifidobacterium	0.21	0.26	0.89	0.11	1.00	0.95	0.47	0.05	0.53	0.84	0.37	0.32	0.63	0.16	0.68	0.79	0.58	0.74	0.42	0.00
Coprobacillus	0.11	0.79	0.95	0.21	0.89	1.00	0.16	0.53	0.00	0.32	0.47	0.58	0.63	0.42	0.74	0.84	0.68	0.05	0.26	0.37
Paraprevotella	0.79	0.53	0.16	0.95	0.47	0.00	0.58	0.63	0.68	0.21	0.74	0.89	0.11	0.84	0.42	0.26	0.05	1.00	0.37	0.32
Mitsuokella	0.79	0.63	0.42	0.47	0.32	0.00	0.95	0.05	0.37	0.16	0.11	0.26	0.84	0.74	1.00	0.68	0.89	0.21	0.53	0.58
Enterobacter	0.32	1.00	0.68	0.63	0.47	0.74	0.21	0.53	0.79	0.26	0.84	0.95	0.05	0.58	0.37	0.11	0.42	0.16	0.89	0.00
Lactobacillus	0.37	0.05	1.00	0.16	0.95	0.47	0.79	0.00	0.63	0.58	0.32	0.89	0.21	0.68	0.74	0.84	0.42	0.53	0.26	0.11

(B)

FAMILY	SAK7	SK5	976AB	LB1	882RK	A1	1660SH	SH3	976DB	683RD	DB2	IS8	199SUBD	U2	SOH4	882AK	1660SK	683PD	AA6	199SD
Veillonellaceae	0.32	0.84	0.05	0.21	0.95	1.00	0.68	0.58	0.11	0.26	0.79	0.63	0.47	0.16	0.89	0.53	0.74	0.42	0.37	0.00
Porphyromonadaceae	0.16	0.53	0.26	0.58	0.05	0.00	0.63	0.79	0.32	1.00	0.84	0.21	0.37	0.95	0.74	0.68	0.11	0.89	0.42	0.47
Desulfovibrionaceae	0.58	0.21	0.11	0.47	0.32	0.84	0.37	0.63	0.53	0.95	0.26	0.42	0.79	0.00	0.74	0.05	0.89	0.68	1.00	0.16
Bacteroidaceae	0.84	0.53	0.16	0.68	0.05	0.00	0.58	0.74	0.32	0.79	0.89	0.37	0.21	0.95	0.63	0.26	0.11	1.00	0.42	0.47
Lactobacillaceae	0.37	0.05	0.95	0.16	1.00	0.47	0.79	0.00	0.84	0.58	0.32	0.89	0.21	0.68	0.74	0.63	0.42	0.53	0.26	0.11
Streptococcaceae	0.53	0.79	1.00	0.84	0.95	0.58	0.26	0.68	0.16	0.00	0.74	0.63	0.05	0.21	0.32	0.89	0.37	0.47	0.11	0.42
Enterobacteriaceae	0.53	0.79	0.47	0.63	0.89	1.00	0.42	0.84	0.00	0.37	0.68	0.58	0.16	0.32	0.21	0.95	0.11	0.26	0.74	0.05
Bifidobacteriaceae	0.21	0.26	1.00	0.11	0.89	0.95	0.47	0.00	0.79	0.84	0.37	0.32	0.63	0.16	0.68	0.53	0.58	0.74	0.42	0.05
Coriobacteriaceae	0.11	0.47	1.00	0.63	0.53	0.89	0.16	0.00	0.84	0.74	0.58	0.21	0.79	0.95	0.26	0.42	0.68	0.32	0.37	0.05
Lachnospiraceae	0.11	0.37	0.58	0.63	0.26	0.00	0.42	0.79	0.32	0.21	0.16	0.89	0.68	0.74	0.47	0.53	1.00	0.95	0.84	0.05
Pasteurellaceae	0.53	0.89	0.95	0.11	1.00	0.00	0.58	0.68	0.26	0.37	0.63	0.79	0.32	0.47	0.21	0.05	0.74	0.42	0.16	0.84
Prevotellaceae	0.53	0.74	0.26	0.84	0.16	0.00	1.00	0.68	0.42	0.32	0.63	0.37	0.11	0.79	0.47	0.58	0.05	0.95	0.21	0.89
Clostridiaceae	0.05	0.32	0.68	0.37	1.00	0.00	0.53	0.47	0.16	0.58	0.26	0.79	0.89	0.11	0.63	0.42	0.95	0.74	0.84	0.21
Succinivibrionaceae	0.11	0.79	0.58	0.42	0.26	0.21	0.16	0.53	1.00	0.95	0.32	0.68	0.63	0.05	0.47	0.74	0.89	0.00	0.84	0.37
Acidaminococcaceae	0.16	0.21	0.42	0.58	0.47	0.53	0.26	0.95	1.00	0.11	0.05	0.37	0.74	0.79	0.32	0.63	0.84	0.00	0.68	0.89
Rikenellaceae	0.00	0.42	0.37	0.63	0.32	0.89	0.47	0.05	0.79	0.58	0.74	0.84	0.68	0.21	0.95	0.26	0.53	0.16	1.00	0.11
Erysipelotrichaceae	0.47	0.32	1.00	0.16	0.95	0.89	0.37	0.00	0.68	0.84	0.05	0.79	0.53	0.63	0.26	0.21	0.74	0.11	0.58	0.42
Eubacteriaceae	0.26	0.21	0.05	0.58	0.37	0.00	0.89	0.47	0.42	0.16	0.63	0.32	0.84	0.79	0.53	0.68	1.00	0.95	0.74	0.11
Ruminococcaceae	0.16	0.42	0.63	0.47	0.21	0.00	0.26	0.53	0.05	0.79	0.32	0.89	0.84	0.11	0.37	0.58	0.95	1.00	0.74	0.68
Sutterellaceae	1.00	0.63	0.26	0.37	0.89	0.05	0.95	0.42	0.84	0.74	0.79	0.32	0.11	0.47	0.58	0.21	0.53	0.00	0.68	0.16
Leuconostocaceae	0.00	0.68	1.00	0.37	0.89	0.95	0.11	0.26	0.53	0.47	0.32	0.84	0.05	0.79	0.58	0.16	0.63	0.74	0.42	0.21

(C)

ORDER	SAK7	SK5	882RK	LB1	976AB	A1	1660SH	SH3	882AK	683RD	DB2	IS8	199SUBD	U2	SOH4	976DB	1660SK	683PD	AA6	199SD
Aeromonadales	0.00	0.79	0.21	0.42	0.58	0.26	0.11	0.53	0.74	0.95	0.32	0.68	0.63	0.05	0.47	1.00	0.89	0.16	0.84	0.37
Erysipelotrichales	0.47	0.32	0.95	0.16	1.00	0.89	0.37	0.00	0.21	0.84	0.05	0.79	0.53	0.63	0.26	0.68	0.74	0.11	0.58	0.42
Desulfovibrionales	0.58	0.21	0.32	0.47	0.16	0.84	0.37	0.63	0.05	0.95	0.26	0.42	0.79	0.00	0.74	0.53	0.89	0.68	1.00	0.11
Pasteurellales	0.53	0.89	1.00	0.11	0.95	0.00	0.58	0.68	0.05	0.37	0.63	0.79	0.32	0.47	0.21	0.26	0.74	0.42	0.16	0.84
Selenomonadales	0.11	0.84	0.95	0.21	0.00	1.00	0.47	0.74	0.53	0.05	0.79	0.42	0.58	0.37	0.89	0.68	0.63	0.16	0.32	0.26
Lactobacillales	0.32	0.74	1.00	0.84	0.95	0.79	0.53	0.58	0.89	0.11	0.63	0.68	0.00	0.37	0.42	0.47	0.21	0.26	0.05	0.16
Enterobacteriales	0.53	0.79	0.89	0.63	0.47	1.00	0.42	0.84	0.95	0.37	0.68	0.58	0.16	0.32	0.21	0.00	0.11	0.26	0.74	0.05
Bifidobacteriales	0.21	0.26	0.89	0.11	1.00	0.95	0.47	0.05	0.53	0.84	0.37	0.32	0.63	0.16	0.68	0.79	0.58	0.74	0.42	0.00
Clostridiales	0.05	0.16	0.74	0.47	0.37	0.00	0.68	0.58	0.53	0.32	0.26	0.79	0.89	0.63	0.42	0.11	1.00	0.95	0.84	0.21
Spirochaetales	0.00	0.32	0.63	0.58	1.00	0.68	0.05	0.21	0.26	0.74	0.47	0.95	0.84	0.16	0.53	0.42	0.89	0.11	0.37	0.79
Coriobacteriales	0.11	0.47	0.53	0.63	1.00	0.89	0.16	0.00	0.42	0.74	0.58	0.21	0.79	0.95	0.26	0.84	0.68	0.32	0.37	0.05
Bacteroidales	0.58	0.74	0.11	0.79	0.26	0.00	0.95	0.63	0.53	0.42	0.68	0.37	0.16	0.89	0.47	0.32	0.05	1.00	0.21	0.84
Burkholderiales	0.95	0.68	0.89	0.42	0.16	0.21	1.00	0.32	0.11	0.58	0.74	0.37	0.47	0.26	0.63	0.84	0.53	0.00	0.79	0.05

(D)

CLASS	SAK7	SK5	882RK	LB1	976AB	A1	1660SH	SH3	882AK	683RD	DB2	IS8	199SUBD	U2	SOH4	976DB	1660SK	683PD	AA6	199SD
Alphaproteobacteria	0.00	0.47	0.11	0.68	0.32	0.79	0.37	0.58	0.21	0.53	0.63	1.00	0.89	0.05	0.84	0.26	0.95	0.16	0.74	0.42
Deltaproteobacteria	0.53	0.26	0.16	0.47	0.11	0.84	0.37	0.63	0.32	0.95	0.21	0.58	0.79	0.00	0.74	0.42	0.89	0.68	1.00	0.05
Gammaproteobacteria	0.47	0.79	0.89	0.63	0.53	1.00	0.32	0.84	0.95	0.37	0.68	0.58	0.05	0.21	0.16	0.42	0.26	0.11	0.74	0.00
Erysipelotrichi	0.47	0.32	0.95	0.16	1.00	0.89	0.37	0.00	0.21	0.84	0.05	0.79	0.53	0.63	0.26	0.68	0.74	0.11	0.58	0.42
Spirochaetia	0.00	0.32	0.63	0.58	1.00	0.68	0.05	0.21	0.26	0.74	0.47	0.95	0.84	0.16	0.53	0.42	0.89	0.11	0.37	0.79
Bacteroidia	0.58	0.74	0.11	0.79	0.26	0.00	0.95	0.63	0.53	0.42	0.68	0.37	0.16	0.89	0.47	0.32	0.05	1.00	0.21	0.84
Negativicutes	0.11	0.84	0.95	0.21	0.00	1.00	0.47	0.74	0.53	0.05	0.79	0.42	0.58	0.37	0.89	0.68	0.63	0.16	0.32	0.26
Clostridia	0.05	0.16	0.74	0.47	0.37	0.00	0.68	0.58	0.53	0.32	0.26	0.79	0.89	0.63	0.42	0.11	1.00	0.95	0.84	0.21
Actinobacteria (class)	0.11	0.26	0.84	0.32	1.00	0.95	0.21	0.05	0.42	0.89	0.47	0.16	0.68	0.53	0.63	0.79	0.58	0.74	0.37	0.00
Bacilli	0.32	0.74	1.00	0.84	0.95	0.79	0.53	0.58	0.89	0.11	0.63	0.68	0.00	0.37	0.42	0.47	0.21	0.26	0.05	0.16
Betaproteobacteria	0.95	0.84	0.79	0.26	0.32	0.58	1.00	0.89	0.11	0.42	0.74	0.21	0.37	0.16	0.47	0.63	0.53	0.00	0.68	0.05

(E)

PHYLUM	SAK7	SK5	882RK	LB1	976AB	A1	1660SH	SH3	882AK	683RD	DB2	IS8	199SUBD	U2	SOH4	976DB	1660SK	683PD	AA6	199SD
Bacteroidetes	1.00	0.58	0.05	0.84	0.26	0.00	0.95	0.63	0.42	0.53	0.68	0.37	0.16	0.79	0.47	0.32	0.11	0.89	0.21	0.74
Fusobacteria	0.84	0.58	0.95	0.26	0.79	1.00	0.11	0.32	0.00	0.21	0.42	0.53	0.63	0.16	0.68	0.89	0.74	0.05	0.37	0.47
Euryarchaeota	0.84	0.21	0.42	0.53	0.16	1.00	0.37	0.26	0.05	0.32	0.58	0.47	0.95	0.00	0.79	0.74	0.89	0.11	0.63	0.68
Actinobacteria	0.32	0.21	0.68	0.37	1.00	0.95	0.11	0.05	0.16	0.84	0.47	0.42	0.89	0.26	0.74	0.63	0.79	0.53	0.58	0.00
Proteobacteria	0.32	0.68	0.89	0.37	0.21	1.00	0.16	0.79	0.95	0.53	0.47	0.58	0.26	0.05	0.42	0.84	0.63	0.00	0.74	0.11
Spirochaetes	0.58	0.11	0.47	0.37	1.00	0.74	0.00	0.21	0.05	0.68	0.32	0.95	0.89	0.16	0.53	0.63	0.79	0.26	0.42	0.84
Synergistetes	0.63	0.37	0.00	0.68	0.11	0.26	0.05	0.58	0.47	0.74	0.42	0.79	1.00	0.16	0.84	0.32	0.89	0.21	0.95	0.53
Firmicutes	0.16	0.37	1.00	0.42	0.89	0.74	0.21	0.26	0.53	0.47	0.11	0.68	0.84	0.00	0.58	0.32	0.95	0.63	0.79	0.05

Table S6.

(A) GENUS

Genus	Score1	Score2	Score3	Overall Score	Power (0.05)	Power (0.01)	Reboot Z-value	P
Dorea	-0.28	-0.07	0.31	-0.03			1.07	
Leuconostoc	-0.44	0.02	0.07	-0.10			0.30	
Dialister	-0.19	0.08	0.13	0.17			0.79	
Succinatimonas	0.04	0.12	0.06	0.21			1.48	
Collinsella	0.02	-0.05	-0.11	0.05			1.42	
Phascolarctobacterium	0.25	0.26	0.09	0.38*	0.31	0.11	2.26	0.012
Veillonella	-0.80	-0.40	-0.05	-0.51	0.99	0.91	-2.50	0.005
Roseburia	0.00	0.42	0.51*	0.46	0.63	0.31	1.66	0.048
Catenibacterium	-0.39	-0.12	0.17	-0.09			0.53	
Prevotella	-0.22	-0.03	0.13	0.01			0.61	
Faecalibacterium	0.38	0.38	0.19	0.49*	0.62	0.30	2.71	0.003
Streptococcus	-0.70*	-0.58	-0.21	-0.60	0.97	0.89	-2.07	0.019
Shigella	-0.62	-0.42	-0.18	-0.50			-1.48	
Escherichia	-0.59*	-0.50	-0.23	-0.58	0.85	0.62	-1.86	0.032
Coprococcus	0.03	0.22	0.14	0.32			1.40	
Butyrivibrio	0.17	0.45	0.38	0.52*	0.63	0.34	1.93	0.027
Eubacterium	0.07	0.37	0.46	0.42			1.55	
Bifidobacterium	-0.17	0.04	-0.20	-0.04			0.91	
Coprobacillus	-0.49	-0.05	-0.13	-0.14			-0.24	
Paraprevotella	-0.49	-0.13	0.25	-0.13			0.77	
Mitsuokella	-0.27	0.08	0.16	0.17			0.70	
Enterobacter	-0.75*	-0.25	0.20	-0.35	0.97	0.89	-1.86	0.032
Lactobacillus	-0.53	0.01	0.16	-0.04			0.57	
G1	0.07	0.46	0.38	0.53*	0.75	0.47	1.82	0.034
G4	-0.98	-0.92	-0.66	-0.99	1.00	1.00	-1.75	0.044

(B) FAMILY

FAMILY	Score1	Score2	Score3	Overall Score	Power (0.05)	Power (0.01)	Reboot Z-value	P
Veillonellaceae	-0.42	-0.19	0.06	-0.30			-1.38	
Porphyromonadaceae	0.32	0.13	0.07	0.22			0.96	
Desulfovibrionaceae	0.31	0.21	-0.09	0.14	0.24	0.49	0.89	
Bacteroidaceae	0.03	0.06	0.16	0.08			0.42	
Lactobacillaceae	-0.25	-0.04	0.10	-0.09			-0.68	
Streptococcaceae	-0.58	-0.58	-0.20	-0.60*	0.76	0.51	-2.25	0.0123
Enterobacteriaceae	-0.56	-0.53	-0.23	-0.62*	0.44	0.21	-2.19	0.0143
Bifidobacteriaceae	0.11	0.01	-0.21	-0.06			-0.61	
Coriobacteriaceae	0.03	-0.05	-0.28	-0.09			-0.85	
Lachnospiraceae	0.22	0.33	0.43	0.37	0.57	0.32	1.25	
Pasteurellaceae	-0.37	-0.25	0.08	-0.22			-1.14	
Prevotellaceae	-0.02	-0.03	0.13	0.01			0.39	
Clostridiaceae	0.21	0.15	0.17	0.21	0.61	0.35	0.63	
Succinivibrionaceae	0.07	-0.06	0.01	0.00			0.25	
Acidaminococcaceae	0.36	0.25	0.02	0.33			1.07	
Rikenellaceae	-0.08	0.17	0.05	0.04			0.45	
Erysipelotrichaceae	-0.12	-0.02	-0.14	-0.08			-0.38	
Eubacteriaceae	0.28	0.37	0.46	0.42	0.61	0.35	1.42	
Ruminococcaceae	0.46	0.34	0.20	0.45	0.68	0.42	1.43	
Sutterellaceae	-0.48	-0.36	0.04	-0.40			-1.33	
Leuconostocaceae	-0.60	-0.37	-0.11	-0.50			-2.09	0.0184

(C) ORDER

ORDER	Score1	Score2	Score3	Overall Score	Power (0.05)	Power (0.01)	Reboot Z-value	P
Aeromonadales	0.07	-0.06	0.01	0.00			0.230	
Erysipelotrichales	-0.12	-0.02	-0.14	-0.08			-0.421	
Desulfovibrionales	0.31	0.21	-0.09	0.14			0.917	
Pasteurellales	-0.37	-0.25	0.08	-0.22			-1.172	
Selenomonadales	-0.23	-0.03	0.09	-0.10			-0.684	
Lactobacillales	-0.67*	-0.60	-0.24	-0.66	0.98	0.93	-2.405	0.0081
Enterobacteriales	-0.56	-0.53	-0.23	-0.62*	0.85	0.65	-2.209	0.0136
Bifidobacteriales	0.11	0.01	-0.21	-0.06			-0.595	
Clostridiales	0.26	0.33	0.37	0.39	0.67	0.41	1.106	
Spirochaetales	-0.21	-0.32	-0.38	-0.29			-1.044	
Coriobacteriales	0.03	-0.05	-0.28	-0.09			-0.847	
Bacteroidales	-0.01	-0.02	0.13	0.03			0.378	
Burkholderiales	-0.44	-0.27	0.05	-0.33			-1.351	

(CLASS)

CLASS	Score1	Score2	Score3	Overall Score	Power (0.05)	Power (0.01)	Reboot Z-value	P
Alphaproteobacteria	-0.18	-0.04	-0.04	-0.11			-0.457	
Deltaproteobacteria	0.24	0.21	-0.01	0.14			0.727	
Gammaproteobacteria	-0.56	-0.50	-0.20	-0.59*	0.79	0.56	-1.962	0.0249
Erysipelotrichi	-0.12	-0.02	-0.14	-0.08			-0.384	
Spirochaetia	-0.21	-0.32	-0.38	-0.29			-1.043	
Bacteroidia	-0.01	-0.02	0.13	0.03			0.379	
Negativicutes	-0.23	-0.03	0.09	-0.10			-0.667	
Clostridia	0.26	0.33	0.37	0.39	0.67	0.41	1.122	
Actinobacteria (class)	0.20	0.08	-0.25	0.01			-0.743	
Bacilli	-0.67	-0.60	-0.25	-0.67	0.98	0.93	-2.414	0.0079
Betaproteobacteria	-0.58*	-0.45	-0.09	-0.56	0.67	0.41	-1.803	0.0357

(F) PHYLUM

PHYLUM	Score1	Score2	Score3	Overall Score	Power (0.05)	Power (0.01)	Reboot Z-value	P
Bacteroidetes	-0.11	-0.13	0.05	-0.10			-0.324	
Fusobacteria	-0.25	-0.14	-0.17	-0.23			-0.773	
Euryarchaeota	0.11	0.22	-0.05	0.16			0.731	
Actinobacteria	0.17	0.12	-0.22	0.05			-0.593	
Proteobacteria	-0.23	-0.18	-0.06	-0.23			-0.689	
Spirochaetes	0.18	0.20	-0.13	0.19			0.622	
Synergistetes	0.35	0.27	0.10	0.35			1.105	
Firmicutes	0.10	0.04	-0.09	0.00			0.308	

Table S7.

(A)

Feature	P-value (uncorrected)	Corrected P-value (<0.1)	Post-hoc test (Tukey-Kramer) [95%CI;P-value<0.05)	Effect size(Eta-squared)
G1	5.75e-4	6.90e-3	AH>SM	0.584
Streptococcus	3.05e-4	7.32e-3	SM>AH,SM>BL	0.614
Escherichia	4.07e-3	0.033	SM>AH	0.477
Shigella	0.013	0.081	SM>AH	0.398
Enterobacter	0.022	0.087	SM>AH	0.363

(B)

Feature	P-value (uncorrected)	Corrected P-value (<0.1)	Effect size (Eta-squared)	Post-hoc test (Tukey-Kramer) [95%CI;P-value<0.05]
Escherichia	3.53e-3	0.028	0.477	SM>AH
Streptococcus	2.59e-3	0.031	0.614	SM>AH,SM>BL
G1	1.56e-3	0.038	0.584	AH>SM
Veillonella	0.015	0.088	0.364	SM>AH
Shigella	0.019	0.089	0.398	SM>AH

Table S8.

COG	Category	Annotation	Z-Score1 (Height for age)	Z-Score2 (Weight for age)	Z-Score3 (Weight for height)	Overall Score	ReBoot Z-Score
COG2852	S	Uncharacterized protein conserved in bacteria	0.65	0.83	0.44	0.80	3.25
COG1148	C	Heterodisulfide reductase, subunit A and related polyferredoxins	0.60	0.67	0.40	0.76	2.88
COG0428	P	Predicted divalent heavy-metal cations transporter	0.53	0.58	0.47	0.69	2.85
COG5001	T	Predicted signal transduction protein containing a membrane domain, an EAL and a GGDEF domain	0.49	0.66	0.55	0.70	2.73
COG2768	R	Uncharacterized Fe-S center protein	0.66	0.58	0.27	0.66	2.67
COG1657	I	Squalene cyclase	0.33	0.82	0.60	0.73	2.66
COG0404	E	Glycine cleavage system T protein (aminomethyltransferase)	0.45	0.69	0.54	0.68	2.61
COG3387	G	Glucoamylase and related glycosyl hydrolases	0.40	0.69	0.40	0.58	2.47
COG3893	L	Inactivated superfamily I helicase	0.40	0.62	0.51	0.65	2.46
COG1787	V	Predicted endonuclease distantly related to archaeal Holliday junction resolvase and Mrr-like restriction enzymes	0.44	0.70	0.49	0.65	2.45
COG1647	R	Esterase/lipase	0.52	0.51	0.19	0.55	2.44
COG4231	C	Indolepyruvate ferredoxin oxidoreductase, alpha and beta subunits	0.65	0.40	0.15	0.54	2.41

COG3320	Q	Putative dehydrogenase domain of multifunctional non-ribosomal peptide synthetases and related enzymes	0.44	0.66	0.45	0.63	2.39
COG1003	E	Glycine cleavage system protein P (pyridoxal-binding), C-terminal domain	0.44	0.57	0.51	0.63	2.37
COG4088	F	Predicted nucleotide kinase	0.37	0.48	0.15	0.42	2.33
COG0782	K	Transcription elongation factor	0.59	0.54	0.19	0.59	2.31
COG3191	EQ	L-aminopeptidase/D-esterase	0.62	0.33	0.03	0.47	2.30
COG3199	S	Uncharacterized conserved protein	0.32	0.62	0.45	0.58	2.30
COG0499	H	S-adenosylhomocysteine hydrolase	0.32	0.50	0.27	0.46	2.27
COG3828	S	Uncharacterized protein conserved in bacteria	0.42	0.59	0.49	0.65	2.26
COG4927	R	Predicted choloylglycine hydrolase	0.66	0.51	-0.06	0.51	2.26
COG4941	K	Predicted RNA polymerase sigma factor containing a TPR repeat domain	0.60	0.63	0.32	0.62	2.25
COG4094	S	Predicted membrane protein	0.06	0.38	0.58	0.33	2.22
COG4747	R	ACT domain-containing protein	0.64	0.54	0.28	0.62	2.21
COG2152	G	Predicted glycosylase	0.39	0.63	0.58	0.65	2.19
COG3267	U	Type II secretory pathway, component ExeA (predicted ATPase)	0.40	0.43	0.38	0.53	2.17
COG1083	M	CMP-N-acetylneuraminic acid synthetase	0.47	0.64	0.44	0.64	2.17
COG1384	J	Lysyl-tRNA synthetase (class I)	0.35	0.65	0.47	0.66	2.16
COG1492	H	Cobyric acid synthase	0.46	0.52	0.35	0.61	2.15
COG1633	S	Uncharacterized conserved protein	0.60	0.59	0.14	0.57	2.15

COG4753	T	Response regulator containing CheY-like receiver domain and AraC-type DNA-binding domain	0.14	0.59	0.64	0.49	2.14
COG3746	P	Phosphate-selective porin	0.32	0.50	0.44	0.53	2.13
COG1228	Q	Imidazolonepropionase and related amidohydrolases	0.37	0.55	0.49	0.60	2.13
COG2114	T	Adenylate cyclase, family 3 (some proteins contain HAMP domain)	0.35	0.56	0.26	0.50	2.12
COG1474	LO	Cdc6-related protein, AAA superfamily ATPase	0.56	0.54	-0.02	0.48	2.12
COG1244	R	Predicted Fe-S oxidoreductase	0.26	0.62	0.40	0.55	2.12
COG1082	G	Sugar phosphate isomerases/epimerases	0.52	0.64	0.38	0.62	2.11
COG2210	S	Uncharacterized conserved protein	0.59	0.36	-0.06	0.41	2.09
COG3883	S	Uncharacterized protein conserved in bacteria	0.35	0.39	0.06	0.40	2.09
COG2158	R	Uncharacterized protein containing a Zn-finger-like domain	0.64	0.51	0.25	0.61	2.07
COG1179	H	Dinucleotide-utilizing enzymes involved in molybdopterin and thiamine biosynthesis family 1	0.40	0.60	0.51	0.63	2.07
COG4359	E	Uncharacterized conserved protein, possibly involved in methylthioadenosine recycling	0.41	0.59	0.30	0.51	2.06
COG3862	S	Uncharacterized protein with conserved CXXC pairs	0.43	0.49	0.34	0.55	2.06
COG0326	O	Molecular chaperone, HSP90 family	0.51	0.58	0.37	0.65	2.05
COG2929	S	Uncharacterized protein conserved in bacteria	0.15	0.53	0.39	0.39	2.05
COG1664	M	Integral membrane protein CcmA involved in cell shape determination	0.47	0.63	0.32	0.57	2.04

COG1060	HR	Thiamine biosynthesis enzyme ThiH and related uncharacterized enzymes	0.12	0.51	0.64	0.47	2.02
COG2836	S	Uncharacterized conserved protein	-0.05	0.49	0.41	0.34	2.02
COG4252	T	Predicted transmembrane sensor domain	0.37	0.58	0.34	0.52	2.02
COG3315	Q	O-Methyltransferase involved in polyketide biosynthesis	0.54	0.57	0.37	0.65	2.01
COG2851	C	H+/citrate symporter	0.65	0.34	0.11	0.46	2.01
COG2899	S	Uncharacterized protein conserved in bacteria	0.46	0.63	0.55	0.70	2.01
COG4898	S	Uncharacterized protein conserved in bacteria	0.54	0.47	0.21	0.58	2.01
COG5616	S	Predicted integral membrane protein	0.58	0.30	-0.27	0.34	2.00
COG4209	G	ABC-type polysaccharide transport system, permease component	0.43	0.53	0.35	0.55	1.98
COG4630	F	Xanthine dehydrogenase, iron-sulfur cluster and FAD-binding subunit A	0.35	0.52	0.26	0.52	1.98
COG0070	E	Glutamate synthase domain 3	0.55	0.47	0.23	0.56	1.97
COG1351	F	Predicted alternative thymidylate synthase	0.29	0.44	0.27	0.46	1.96
COG1724	N	Predicted periplasmic or secreted lipoprotein	0.54	0.35	0.11	0.48	1.95
COG1061	KL	DNA or RNA helicases of superfamily II	0.35	0.42	0.44	0.53	1.95
COG1963	S	Uncharacterized protein conserved in bacteria	0.41	0.49	0.27	0.47	1.95
COG2602	V	Beta-lactamase class D	0.23	0.54	0.56	0.56	1.93
COG1908	C	Coenzyme F420-reducing hydrogenase, delta subunit	0.61	0.56	0.28	0.67	1.92
COG0338	L	Site-specific DNA methylase	0.54	0.36	0.08	0.42	1.92

Table S9.

COG	Category	Annotation	Z-Score1 (Height for age)	Z-Score2 (Weight for age)	Z-Score3 (Weight for height)	Overall Score	ReBoot Z-Score
COG4481	S	Uncharacterized protein conserved in bacteria	-0.18	-0.52	-0.76	-0.55	-3.55
COG0681	U	Signal peptidase I	-0.58	-0.72	-0.26	-0.66	-3.35
COG2960	S	Uncharacterized protein conserved in bacteria	-0.63	-0.60	-0.24	-0.71	-3.11
COG0569	P	K+ transport systems, NAD-binding component	-0.16	-0.64	-0.51	-0.48	-2.87
COG4940	U	Competence protein ComGF	-0.50	-0.67	-0.47	-0.69	-2.86
COG3647	S	Predicted membrane	-0.54	-0.49	-0.22	-0.59	-2.80
COG3104	E	Dipeptide/tripeptide	-0.60	-0.67	-0.37	-0.73	-2.77
COG4924	S	Uncharacterized protein conserved in bacteria	-0.27	-0.68	-0.58	-0.67	-2.73
COG2893	G	Phosphotransferase system, mannose/fructose-specific component IIA	-0.56	-0.56	-0.30	-0.63	-2.73
COG3965	P	Predicted Co/Zn/Cd cation transporters	-0.62	-0.25	0.07	-0.37	-2.71
COG3522	S	Uncharacterized protein conserved in bacteria	-0.56	-0.51	-0.19	-0.60	-2.68
COG3700	R	Acid phosphatase (class B)	-0.41	-0.64	-0.52	-0.68	-2.68
COG3455	S	Uncharacterized protein conserved in bacteria	-0.59	-0.61	-0.33	-0.70	-2.66
COG4469	R	Competence protein	-0.55	-0.53	-0.24	-0.60	-2.66
COG4133	O	ABC-type transport system involved in cytochrome c biogenesis, ATPase	-0.62	-0.60	-0.27	-0.68	-2.66
COG5571	N	Autotransporter protein or domain, integral membrane beta-barrel involved in	-0.61	-0.54	-0.21	-0.63	-2.60
COG4215	E	ABC-type arginine transport system, permease	-0.53	-0.54	-0.26	-0.62	-2.57
COG1025	O	Secreted/periplasmic Zn-dependent peptidases,	-0.41	-0.50	-0.28	-0.58	-2.57
COG2814	G	Arabinose efflux permease	-0.53	-0.62	-0.32	-0.67	-2.56
COG4150	P	ABC-type sulfate transport system, periplasmic	-0.44	-0.52	-0.36	-0.57	-2.55
COG2044	R	Predicted peroxiredoxins	-0.71	-0.56	-0.18	-0.69	-2.54
COG3539	NU	P pilus assembly protein,	-0.57	-0.49	-0.16	-0.60	-2.51
COG4128	R	Zonula occludens toxin	-0.59	-0.41	-0.12	-0.57	-2.51

COG0271	T	Stress-induced morphogen (activity unknown)	-0.60	-0.38	-0.02	-0.44	-2.50
COG3154	I	Putative lipid carrier protein	-0.61	-0.47	-0.12	-0.58	-2.49
COG3196	S	Uncharacterized protein conserved in bacteria	-0.60	-0.36	0.01	-0.48	-2.48
COG2514	R	Predicted ring-cleavage extradiol dioxygenase	-0.57	-0.59	-0.29	-0.66	-2.46
COG0387	P	Ca2+/H+ antiporter	-0.41	-0.49	-0.29	-0.54	-2.44
COG3499	R	Phage protein U	-0.45	-0.45	-0.25	-0.53	-2.42
COG0778	C	Nitroreductase	-0.39	-0.59	-0.26	-0.51	-2.42
COG3121	NU	P pilus assembly protein, chaperone PapD	-0.54	-0.51	-0.21	-0.58	-2.41
COG1133	I	ABC-type long-chain fatty acid transport system, fused permease and ATPase	-0.56	-0.37	-0.03	-0.47	-2.40
COG0477	GEPR	Permeases of the major facilitator superfamily	-0.53	-0.61	-0.35	-0.68	-2.40
COG4220	L	Phage DNA packaging protein, Nu1 subunit of	-0.59	-0.49	-0.12	-0.60	-2.39
COG3015	MP	Uncharacterized lipoprotein NlpE involved in copper	-0.51	-0.46	-0.25	-0.57	-2.36
COG2916	R	DNA-binding protein H-NS	-0.48	-0.54	-0.33	-0.60	-2.36
COG4640	S	Predicted membrane	-0.41	-0.51	-0.38	-0.51	-2.35
COG2367	V	Beta-lactamase class A	-0.66	-0.38	-0.09	-0.52	-2.31
COG4943	T	Predicted signal transduction protein	-0.52	-0.49	-0.19	-0.56	-2.31
COG3055	S	Uncharacterized protein conserved in bacteria	-0.46	-0.59	-0.31	-0.58	-2.30
COG3109	T	Activator of osmoprotectant transporter ProP	-0.46	-0.52	-0.29	-0.61	-2.30
COG3061	M	Cell envelope opacity-associated protein A	-0.48	-0.49	-0.21	-0.54	-2.28
COG1621	G	Beta-fructosidases (levanase/invertase)	-0.53	-0.55	-0.12	-0.51	-2.28
COG3456	T	Uncharacterized conserved protein, contains FHA	-0.46	-0.62	-0.38	-0.63	-2.28
COG3232	E	5-carboxymethyl-2-hydroxymuconate isomerase	-0.58	-0.34	0.00	-0.48	-2.27
COG5281	S	Phage-related minor tail	-0.53	-0.48	-0.15	-0.55	-2.27
COG4537	U	Competence protein ComGC	-0.57	-0.52	-0.25	-0.64	-2.26
COG5655	L	Plasmid rolling circle replication initiator protein and truncated derivatives	-0.47	-0.51	-0.41	-0.64	-2.26
COG3720	P	Putative heme degradation	-0.43	-0.58	-0.31	-0.52	-2.26
COG3477	S	Predicted periplasmic/secreted	-0.57	-0.49	-0.18	-0.57	-2.26
COG3256	P	Nitric oxide reductase large	-0.66	-0.28	0.12	-0.46	-2.25

COG3692	S	Uncharacterized protein conserved in bacteria	-0.57	-0.55	-0.22	-0.65	-2.24
COG3161	H	4-hydroxybenzoate synthetase (chorismate	-0.45	-0.44	-0.23	-0.54	-2.24
COG3137	M	Putative salt-induced outer membrane protein	-0.32	-0.51	-0.37	-0.58	-2.23
COG2706	G	3-carboxymuconate cyclase	-0.53	-0.62	-0.37	-0.60	-2.23
COG4571	M	Outer membrane protease	-0.56	-0.61	-0.10	-0.61	-2.23
COG3311	K	Predicted transcriptional	-0.47	-0.46	-0.23	-0.56	-2.21
COG3697	HI	Phosphoribosyl-dephospho-CoA transferase (holo-ACP	-0.09	-0.46	-0.60	-0.47	-2.21
COG4733	S	Phage-related protein, tail component	-0.61	-0.54	-0.19	-0.62	-2.20
COG5464	S	Uncharacterized conserved	-0.43	-0.56	-0.43	-0.58	-2.18
COG2269	J	Truncated, possibly inactive, lysyl-tRNA synthetase (class	-0.53	-0.39	-0.10	-0.45	-2.17
COG2959	H	Uncharacterized enzyme of heme biosynthesis	-0.51	-0.52	-0.26	-0.59	-2.17
COG3425	I	3-hydroxy-3-methylglutaryl CoA synthase	-0.58	-0.51	-0.20	-0.55	-2.16
COG3579	E	Aminopeptidase C	-0.27	-0.57	-0.38	-0.50	-2.16
COG2193	P	Bacterioferritin (cytochrome	-0.01	-0.32	-0.48	-0.30	-2.15
COG3769	R	Predicted hydrolase (HAD superfamily)	-0.43	-0.53	-0.42	-0.59	-2.15
COG1272	R	Predicted membrane protein, hemolysin III	-0.34	-0.54	-0.20	-0.48	-2.15
COG3086	T	Positive regulator of sigma E	-0.38	-0.42	-0.25	-0.51	-2.15
COG0179	Q	2-keto-4-pentenoate hydratase/2-oxohepta-3-ene-1,7-dioic acid hydratase	-0.47	-0.52	-0.03	-0.48	-2.14
COG0596	R	Predicted hydrolases or acyltransferases (alpha/beta hydrolase superfamily)	-0.63	-0.57	-0.25	-0.64	-2.13
COG3151	S	Uncharacterized protein conserved in bacteria	-0.65	-0.53	-0.14	-0.63	-2.13
COG3272	S	Uncharacterized conserved	-0.39	-0.37	-0.17	-0.44	-2.12
COG3520	S	Uncharacterized protein conserved in bacteria	-0.40	-0.44	-0.21	-0.49	-2.12
COG3819	S	Predicted membrane	-0.69	-0.45	-0.12	-0.59	-2.12
COG4533	R	ABC-type uncharacterized transport system,	-0.50	-0.51	-0.26	-0.56	-2.12
COG5455	S	Predicted integral membrane protein	-0.43	-0.42	-0.20	-0.52	-2.11
COG3085	S	Uncharacterized protein conserved in bacteria	-0.64	-0.16	0.13	-0.32	-2.11
COG4138	H	ABC-type cobalamin transport system, ATPase	-0.53	-0.53	-0.22	-0.62	-2.11

COG3316	L	Transposase and inactivated derivatives	-0.56	-0.44	-0.11	-0.55	-2.11
COG1658	L	Small primase-like proteins (Toprim domain)	-0.52	-0.51	-0.27	-0.55	-2.11
COG4950	S	Uncharacterized protein conserved in bacteria	-0.48	-0.47	-0.20	-0.56	-2.11
COG0693	R	Putative intracellular protease/amidase	-0.62	-0.52	-0.22	-0.56	-2.10
COG1280	E	Putative threonine efflux	-0.39	-0.42	-0.16	-0.48	-2.09
COG3149	U	Type II secretory pathway, component PulM	-0.62	-0.43	-0.11	-0.60	-2.09
COG1444	R	Predicted P-loop ATPase fused to an	-0.45	-0.45	-0.20	-0.52	-2.08
COG2128	S	Uncharacterized conserved	-0.45	-0.46	-0.32	-0.52	-2.07
COG3355	K	Predicted transcriptional	-0.47	-0.36	-0.16	-0.48	-2.06
COG5512	R	Zn-ribbon-containing, possibly RNA-binding protein and truncated derivatives	-0.60	-0.45	-0.17	-0.60	-2.05
COG3683	R	ABC-type uncharacterized transport system,	-0.57	-0.34	-0.05	-0.43	-2.05
COG5339	S	Uncharacterized protein conserved in bacteria	-0.49	-0.43	-0.18	-0.50	-2.05
COG3709	P	Uncharacterized component of phosphonate metabolism	-0.47	-0.31	-0.04	-0.43	-2.04
COG2901	KL	Factor for inversion stimulation Fis,	-0.63	-0.37	-0.09	-0.49	-2.04
COG3087	D	Cell division protein	-0.45	-0.37	-0.15	-0.44	-2.04
COG3624	P	Uncharacterized enzyme of phosphonate metabolism	-0.42	-0.45	-0.26	-0.54	-2.03
COG4778	P	ABC-type phosphonate transport system, ATPase	-0.53	-0.45	-0.16	-0.56	-2.03
COG3454	P	Metal-dependent hydrolase involved in phosphonate metabolism	-0.54	-0.38	0.01	-0.47	-2.02
COG4671	R	Predicted glycosyl	-0.34	-0.54	-0.26	-0.45	-2.02
COG3850	T	Signal transduction histidine kinase, nitrate/nitrite-	-0.47	-0.48	-0.24	-0.52	-2.02
COG2198	T	FOG: HPt domain	-0.58	-0.49	-0.15	-0.57	-2.02
COG0376	P	Catalase (peroxidase I)	-0.52	-0.52	-0.23	-0.59	-2.01
COG2261	S	Predicted membrane	-0.64	-0.52	-0.24	-0.66	-2.01
COG4672	S	Phage-related protein	-0.61	-0.42	-0.01	-0.52	-2.00
COG1940	KG	Transcriptional regulator/sugar kinase	-0.53	-0.60	-0.27	-0.60	-2.00
COG0513	LKJ	Superfamily II DNA and RNA helicases	-0.23	-0.54	-0.40	-0.52	-2.00
COG4892	R	Predicted heme/steroid binding protein	0.06	-0.26	-0.46	-0.23	-2.00

COG3778	S	Uncharacterized protein conserved in bacteria	-0.65	-0.58	-0.18	-0.64	-2.00
COG5383	S	Uncharacterized protein conserved in bacteria	-0.55	-0.42	-0.09	-0.52	-2.00
COG1670	J	Acetyltransferases, including N-acetylases of ribosomal	-0.45	-0.41	-0.09	-0.45	-1.99
COG1914	P	Mn2+ and Fe2+ transporters of the NRAMP family	-0.59	-0.51	-0.20	-0.58	-1.99
COG0771	M	UDP-N-acetylmuramoylalanine-D-	-0.52	-0.46	0.05	-0.39	-1.99
COG3013	S	Uncharacterized conserved	-0.49	-0.46	-0.21	-0.56	-1.97
COG0770	M	UDP-N-acetylmuramyl pentapeptide synthase	-0.24	-0.53	-0.34	-0.45	-1.97

Table S10.

Group1	Group2	Group3	Group4	Group5	Group6	Group7	Group8	Group9
GH24	GT55	PL13	GH44	GH101	GT4	GH85	CE7	GH127
GH128	GT9	GH91	GH12	GT22	GH36	GT11	PL11	CE1
PL12	GT77	GH15	GH58	GT43	GT2	GH123	GH35	GH8
PL15	GH104	GH76	PL3	GT25	GT28	GH108	CE12	GH64
GT17	GT73	GT23	GH116	GH23	GT35	PL17	GH30	CE3
GH14	GT52	GH63	CE13	GH4	GT5	GT72	GH73	GT82
GH48	PL14	GH50	GT39	GT74	CE4		GH16	GH98
GT83	GH17	GT26	GH74	GT60	GH5			CE11
GH113	GH19	GH79	GT10	GH13	GH77			GT19
GH114	GH53	PL21	GH47	GT13	GH26			PL1
GH112	GH1	GH75	GT48	GT76	GH32			PL10
GH39	GT14	GH33	CE15	CE14	PL9			GH88
GH6	GH25	GH62	GT6	GH38	GH66			GH105
GH9	GT1	GH117	PL7	GT31				CE8
GT32	GT84	PL22		GH11				GH92
GH93	GH65	GT87		GT80				GH130
PL8	CE9	GT81		GH103				GH29
GH99	GH42	GH110		GT56				GH31
CE2	GT44			GH37				GH94
GT62	GH102			GT20				GT3
GT66	GT51			CE10				GH3
GT42				GT68				GH57
GT71				GT90				GH78
GH71				GT57				GH106
GH86				GT29				GH115
GH87				GH129				CE6
GH120				GH45				GT30
GH121				GT91				GH28
GH70				GH18				GH2
GH68				GT34				GH10
GT47				GT24				GH67
				GH55				GH95
				GT64				GH97
				GT7				GH43
				GT33				GH51
				GT27				GH72
				GT41				GH27
				GH59				GH89
				GT8				GH125
				GH81				GH84
				GT69				GH20
				GT61				GH109
				GT54				
				GT49				
				GT18				
				GT16				
				GT12				
				GH56				

Table S11.

CAZy Groups	Nutritional Indices				SIGNIFICANCE
	SCORE1	SCORE2	SCORE3	OVERALL	
Group_1	0.069	0.216	0.255	0.220	
Group_2	-0.131	-0.105	0.058	-0.086	
Group_3	0.259	0.492	0.335	0.457	STRONG
Group_4	0.117	0.132	0.011	0.117	
Group_5	-0.223	-0.168	-0.059	-0.209	
Group_6	0.237	0.331	0.303	0.376	MODERATE
Group_7	0.039	0.259	0.447	0.299	MODERATE
Group_8	0.226	0.278	0.219	0.316	MODERATE
Group_9	-0.109	-0.212	-0.091	-0.173	
Group_3_6_7**	0.276	0.558	0.559	0.583	STRONG
